# AI-supported early autism identification as public health infrastructure: economic implications for the United States using a scenario-based economic model

**DOI:** 10.3389/fpubh.2026.1886106

**Published:** 2026-08-17

**Authors:** Tannista Banerjee, Arnab Nayak

**Affiliations:** 1 Department of Economics, Auburn University, Auburn, AL, United States; 2 Stetson-Hatcher School of Business, Mercer University, Macon, GA, United States

**Keywords:** artificial intelligence, autism spectrum disorder, diagnostic equity, digital public health, early identification, early intervention, health economics, healthcare resource allocation

## Abstract

**Introduction:**

Artificial intelligence (AI)-supported tools may shorten autism spectrum disorder (ASD) identification pathways, but their public health value depends on whether earlier identification is followed by timely intervention.

**Methods:**

This study developed a calibrated, scenario-based cost-of-illness model to examine how AI-supported early autism identification could affect the timing and magnitude of US societal ASD-related costs under explicitly stated assumptions. The model reconstructed a business-as-usual baseline from 2011 cost cells, calibrated annual per-person cost growth to reproduce a published 2025 national burden benchmark of approximately $461 billion, and projected costs from 2025 to 2050 using a cohort-based stock model. AI-supported tools were represented as accelerators within screening, triage, referral, and clinician-led diagnostic pathways.

**Results:**

Under the Base deployment scenario, cumulative discounted net cost remained positive through 2050 at approximately $52 billion, while annual net costs declined sharply toward zero. The High deployment scenario reached annual net savings by the mid-2040s. Extended-horizon analysis indicated that cumulative fiscal payback may emerge after the primary 25-year policy window. Prevalence, per-person cost growth, and population growth mainly scaled national dollar totals; discounting changed the present value assigned to delayed savings; and the adult trajectory-shift assumption strongly influenced payback timing.

**Discussion:**

These findings are conditional projections, not estimates of realized savings from observed AI deployment. Their interpretation depends on timely intervention access, adequate service capacity, equitable implementation, and durable reductions in downstream support needs. Calibration provides a transparent published baseline for scenario analysis; it does not validate future projections.

## 1 Introduction

Autism spectrum disorder (ASD) is a lifelong neurodevelopmental condition associated with substantial costs for people with autism, their families, and public systems. These costs span healthcare, education, long-term support, and productivity (1–3). US surveillance indicates that the measured prevalence among 8-year-old children increased from one in 150 in 2000 to one in 31 in 2022. This rise reflects changes in diagnostic criteria, awareness, screening, access, and case ascertainment, as well as possible changes in occurrence (4–6). Building on Buescher's 2011 estimate, Leigh and Du (7) projected a US societal burden of $461 billion in 2025. Their estimate extends through adult support needs and productivity losses, making timely and equitable identification a public health concern (8).

A central US public health bottleneck is the delay between initial developmental concern and formal autism diagnosis. Traditional pathways often depend on specialist or multidisciplinary assessment. Data from the Centers for Disease Control and Prevention (CDC) Pathways Survey indicate an average interval of approximately 3 years between parents' first concerns and diagnosis. The majority of children are still diagnosed after age 3, even though concerns often arise before age two (9–11). Access also remains uneven across socioeconomic, racial, ethnic, and geographic groups because qualified evaluators are scarce and diagnostic queues are long (12–14). In a retrospective study of commercially insured US children younger than 6 years, a longer time from the first documented autism-related concern to diagnosis was associated with more healthcare visits and approximately twofold higher all-cause medical costs in the year before diagnosis (15). These clinical, access, and short-run cost consequences create the need to evaluate whether the selected stages of the diagnostic pathway can be shortened safely and equitably.

Artificial intelligence (AI)-supported tools are increasingly being used to reduce delays and inefficiencies at several points in autism identification pathways. They can organize screening information, prioritize referrals, identify children who may benefit from expedited evaluation, and support clinician decision-making. They do not replace comprehensive clinical assessment. Prospective studies suggest that these functions are feasible in practice. Canvas Dx, a U.S. Food and Drug Administration (FDA)-authorized clinical-support device, generated determinate outputs for a subset of young children and directed uncertain cases to further professional evaluation (16). The SenseToKnow app added digitally measured behavioral information to caregiver screening during well-child visits (17).

Eye-tracking tools provide another example. EarliPoint is FDA-cleared as an aid for qualified clinicians in the diagnosis and assessment of autism. Its current indication covers children aged 16–95 months who are at risk based on concerns raised by a parent, caregiver, or health professional (10, 18). Canvas Dx has also been integrated into the autism primary-care workflow of the Extension for Community Healthcare Outcomes program. That study reported feasible integration and shorter observed diagnostic timing than publicly reported specialist waits (19).^1^ Recent reviews document the expanding use of behavioral, questionnaire, video, eye-tracking, and multimodal data for autism screening and diagnostic support (20–22). Taken together, this evidence supports the plausibility that AI can accelerate the selected stages of the identification pathway. It therefore motivates an examination of the long-term public health and economic implications if such acceleration leads to earlier identification.^2^

Earlier identification can create long-term public health and economic value only when it changes access to effective services. The pathway is contingent rather than automatic. Earlier identification may enable earlier intervention, and effective intervention may improve cognitive, language, adaptive, and social-communication outcomes. Sustained gains may then reduce later educational, medical, residential-support, and caregiver burdens. Trials and reviews of the Early Start Denver Model (ESDM), Early Intensive Behavioral Intervention (EIBI), and related approaches report improvements in several developmental domains. Economic studies also identify potential school-age service offsets and longer-run savings when developmental gains persist and translate into lower support needs (9, 23–25). These findings do not establish that earlier identification alone improves outcomes or that every child follows the same trajectory. They support a conditional economic pathway whose effects depend on timely and effective intervention, adequate service capacity, sustained developmental gains, and durable reductions in downstream support needs.

Existing studies establish important parts of this pathway but do not bring them together in a single national economic framework. Leigh and Du (7) provide a national burden framework. Other studies examine diagnostic delay (12, 15), AI-supported assessment (16, 17, 19), early-intervention outcomes (23, 24), or particular cost categories (3, 26). The literature reviewed for this study did not identify a calibrated US scenario model that integrates AI-supported pathway acceleration, time-varying pathway reach, cohort aging, intervention-dependent trajectories, national societal costs, stakeholder-burden distribution, and payback timing. This integration gap limits the ability of policymakers and health systems to examine when, for whom, and under what service-capacity assumptions earlier identification could change long-run costs.

Against this background, this study contributes a transparent, calibrated scenario framework for tracing how alternative autism-identification pathways could change national costs over time. The deployment scenarios determine how quickly AI-supported identification reaches children and how many enter an earlier-identification pathway each year. Population growth and cohort aging determine the size and age distribution of the population incurring costs during each period. Intervention timing and outcome assumptions determine whether earlier identification produces only additional near-term service costs or also produces later reductions in educational, medical, caregiver, and adult-support costs. Calibration translates these pathways into national cost estimates. It shows when costs and savings arise, how burdens are allocated across broad stakeholder groups, and how the results change when uncertain assumptions are varied. By making these assumptions explicit, the analysis supports public health planning under the stated conditions rather than estimating the observed causal effects of current AI deployment.

Frazier et al. (9) provide the closest economic comparator. Like this study, Frazier et al. examine the economic value of scalable diagnostic support linked to earlier intervention. Frazier et al. estimate per-child lifetime savings and annualized national savings implied by reductions in special-education, medical, and residential-care costs. In contrast, this study embeds these cost-offset channels within a calibrated national burden model. It follows successive birth cohorts, allows the net pathway reach to change over time, reports annual and cumulative costs and payback timing, and provides an illustrative stakeholder-burden distribution. Therefore, the present contribution extends the per-child cost-benefit literature into a dynamic national scenario framework.

Accordingly, the objective of this study is to estimate how AI-supported earlier autism identification could change US societal costs from 2025 to 2050 under alternative implementation and outcome pathways. The analysis compares low-, base-, and high-deployment scenarios that differ in how quickly children enter an earlier-identification pathway. It then examines how these pathways interact with intervention timing, demographic change, stakeholder-burden distribution, and uncertainty. Extended payback analyses continue through 2060 and 2070. The framework applies broadly to gaze-based, video-based, mobile, and multimodal AI-supported pathways rather than to any single commercial product. It does not estimate the realized effects from the observed deployment. Instead, it shows the cost implications that follow if earlier identification is connected to timely intervention and durable changes in later support needs.

## 2 Materials and methods

### 2.1 Model logic and evidence foundations

The model developed in Section 2.2 begins by recreating a selected national baseline for autism-related costs under existing patterns of identification, intervention, and support. It then follows successive birth cohorts as they grow older. For each cohort, the model varies how many children with autism reach an earlier-identification pathway, how early that occurs, how costs may change during childhood and school years, and whether some benefits may persist into adulthood. The baseline is calibrated to published national cost estimates. The pathway levers are specified transparently as literature-informed scenario assumptions and are examined through bundled scenarios and one-way sensitivity analyses. They are not observed values from real-world national AI deployment. The model adds costs across ages and years and compares the earlier-identification pathway with business as usual.

The first step in this process is to recreate the national baseline. This step draws on the autism cost-of-illness literature. Autism-related costs extend beyond pediatric healthcare to education, family caregiving, lost productivity, and adult residential and support services. Their composition also changes with age and intellectual-disability status. Life-course societal estimates therefore provide a more suitable foundation than pediatric medical-cost studies alone (2, 3, 7, 27). This literature anchors the all-age cost framework and the published benchmark used for calibration.

For the next step, the model examines how costs may change during childhood and school years. This step draws on the early-intervention and economic literatures. Earlier assessment and intervention can increase near-term spending. Effective intervention may later reduce some education, healthcare, caregiver, and support costs. Studies of applied behavior analysis, EIBI, and related developmental approaches document childhood outcomes, including recent individual-participant evidence of clinically significant but heterogeneous response (23, 24, 28, 29). Economic studies examine intervention costs and later service offsets (25, 30). This evidence anchors the direction of childhood and school-age cost adjustments. This evidence does not imply that earlier identification automatically creates savings.

The long-run step asks whether some benefits persist into adulthood. This question draws on a limited body of evidence. Historical intervention studies document substantial but heterogeneous childhood responses (31–33). Contemporary reviews also document wide variation in adult outcomes and continuing support needs (34). Cost-benefit studies examine how persistent gains could affect later service use and lifetime costs (9, 35). A recent methodological study explains why linking short-term intervention outcomes to long-term economic effects remains difficult (36). However, this literature does not directly establish adult independence or reductions in adult residential costs. Instead, it provides a context for testing alternative long-term pathways under clearly stated assumptions.

The remaining pathway questions concern how many children reach earlier identification and how early they do so. These questions draw on the diagnostic-support and implementation literatures. Prospective studies show that AI-supported tools can assist in screening, triage, referral, and clinician-led assessment, while uncertain cases continue to require professional evaluation (10, 16, 17, 19). Workforce and access studies also show that technical availability does not guarantee completed evaluation or timely intervention (11, 37, 38). This evidence anchors the pathway reach and timing assumptions in the model. It also explains why the model uses alternative deployment scenarios rather than a single forecast. Section 2.2 formalizes each part of this process and cites the specific evidence where it enters the model.

### 2.2 Model specification and calibration

#### 2.2.1 Baseline business-as-usual cost framework

This analysis adopts and extends Leigh and Du's (7) cost-of-illness framework. Their framework builds on Buescher's 2011-dollar estimates of ASD-related costs (2). This reference pathway is termed “business as usual” (BAU).

Individuals with ASD are divided into six cells. The cells combine three age groups (0–5, 6–17, and 18+) with intellectual disability (ID) status. Let d ∈D={ID,NoID} index ID status. For each calendar year *t*, the total national ASD-related cost is


$$\begin{array}{l} \mathrm{TotalCos}{\text{t}}_{\text{t}}^{\text{BAU}}=\underset{\text{a}}{\sum }\underset{\text{d}\in D}{\sum }{\text{N}}_{\text{a},\text{d},\text{t}}\times {\text{B}}_{\text{k}\left(\text{a}\right),\text{d},\text{t}}, \end{array}$$ (1)

where *a* denotes single-year age. The function *k*(*a*)∈{0 − 5, 6 − 17, 18+} maps each age to its cost band. The term *N*_*a, d, t*_ is the number of individuals with ASD at age *a* and ID status *d* in year *t*. The term *B*_*k*(*a*), *d, t*_ is the mean annual cost per person in that cell.

The model retains the 40% with-ID and 60% without-ID splits used by Buescher et al. and Leigh and Du. This split is close to the 37.9% with ID reported in the 2020 Autism and Developmental Disabilities Monitoring (ADDM) surveillance year. This comparison is limited to children with ASD for whom cognitive-ability data were available (2, 5, 7).

Per-person costs in each cell grow at an annual rate *g*:


$$\begin{array}{l} B_{k,d,t}=B_{k,d,2011}\times {\left(1+g\right)}^{t-2011}. \end{array}$$ (2)

Here, *B*_*k, d*, 2011_ is the Buescher et al. cell mean in 2011 US dollars. The rate *g* is a common per-person cost-growth factor applied to medical, non-medical, educational, residential and support, and productivity costs. This common rate is an aggregate simplifying assumption and not a claim that every category follows the same observed growth path. Its base value is calibrated below rather than being selected in advance. The sensitivity bounds are specified after the calibrated value is derived.

The Buescher cells fit the all-age and multicomponent structure required here. More recent Medical Expenditure Panel Survey estimates provide valuable pediatric healthcare comparisons, but they do not include the full set of education, support, and productivity costs used in the national calibration (3, 26).

##### 2.2.1.1 Maximum age and age distribution

The model tracks individuals from age 0 through age 64. This boundary approximates a life-course horizon through the mid-60s and is consistent with age-truncated structures used in several autism lifetime-cost studies. Buescher et al. and Leigh and Du aggregate all adults into an 18+ group rather than modeling single-year adult ages. Ganz (1), however, reports age-specific costs through the 63–66 age group.

The age ceiling does not imply that autism-related costs cease after age 64. Zhao et al. (8) show that later-life costs remain economically important, particularly adult-care and direct non-medical costs for individuals with ASD and ID. Therefore, age 64 is treated as a conservative primary boundary, with an older-age ceiling used only as an auxiliary robustness check.

##### 2.2.1.2 Calibration of the cost growth rate

The parameter *g* is calibrated to reproduce Leigh and Du's rounded 2025 national total of approximately $461 billion. This step reproduces a selected baseline. It does not independently validate the model or its projections after 2025.

The calibration equation is


$$\begin{array}{l} \underset{k}{\sum }\underset{d\in D}{\sum }N_{k,d,2025}\times B_{k,d,2011}\times {\left(1+g\right)}^{14}=461\times 10^{9}. \end{array}$$ (3)

The term *N*_*k, d*, 2025_ applies the Buescher age-band shares and the 40/60 ID split to the modeled 2025 ASD population. The base case uses 1.1% prevalence and a US resident population of approximately 342 million, rounded from the U.S. Census Bureau's July 2025 estimate of 341.8 million (39).

All terms except (1+*g*)^14^ are fixed after the population, prevalence, ID split, age-band shares, and 2011 cost cells are specified. Equation 3 therefore has the closed-form solution


$$\begin{array}{l} g={\left(\frac{461\times 10^{9}}{K}\right)}^{1/14}-1, \end{array}$$ (4)

where


$$\begin{array}{l} K=\underset{k}{\sum }\underset{d\in D}{\sum }N_{k,d,2025}\times B_{k,d,2011}. \end{array}$$

The quantity *K* is the 2025 cost aggregate evaluated at 2011 prices. The Buescher age shares and cost cells give *K*≈*$*251.0 billion. The resulting value is *g*≈4.44% per year.

As a consistency check, an effective rate of approximately 3.3% for 2011–2015 approximately reproduces Leigh and Du's 2015 base-case estimate of $268.3 billion. The analysis uses 3.5% as a rounded lower sensitivity bound near the consistency-check rate and 5.5% as an upper bound, approximately one percentage point above the calibrated 4.44% rate. These values are structured sensitivity bounds and not confidence limits.

##### 2.2.1.3 Cohort-based stock model for prevalence and aging

For years after 2025, individuals age forward 1 year at a time, and new age-0 entrants are added to maintain the selected prevalence. The model begins with an estimated US resident population of approximately 342 million in 2025 (39). Population grows at 0.2% per year in the base case.

This rate is a simple constant-growth approximation to the Congressional Budget Office (CBO) September 2025 long-run outlook. The CBO projects a broader Social Security area population to grow from 350 million in 2025 to 367 million in 2055. The constant compound rate connecting those endpoints is approximately 0.16%. The CBO also describes its year-by-year projection as averaging approximately 0.2% annual growth over the period. The two figures are consistent because 0.2% is a rounded description of the projected annual path, whereas 0.16% is the exact constant rate connecting the endpoints. Although deaths are projected to exceed births beginning in 2031, positive net immigration is expected to continue, supporting overall population growth (40).

The population concepts and projection methods are not identical; therefore, the comparison is directional rather than a direct level match. The analysis also examines 0.5% annual growth as a higher-growth sensitivity.^3^ This sensitivity tests how rapid population growth changes the national cost levels without changing the economic structure of the model.

The base case retains Leigh and Du's 1.1% prevalence rate. This choice preserves comparability with the reference model. The CDC ADDM Network reported prevalence estimates of 2.78% among 8-year-old children in 2020 and 3.23% in 2022 (5, 6). These values are used as higher-prevalence burden-scaling cases. They are not treated as direct estimates of the national prevalence for all ages.

Let *A*_max_ = 64 denote the maximum modeled age. Let d ∈D index ID status. The ASD stock evolves in two steps.

First, each individual below the age ceiling ages by 1 year:


$$\begin{array}{l} N_{a,d,t}=N_{a-1,d,t-1}\;for\;a=1,\ldots ,A_{\max}. \end{array}$$ (5)

Individuals who would age beyond *A*_max_ exit the modeled stock. This step shifts the age distribution forward. It also increases the adult cost-band share as cohorts age.

Second, new age-0 entrants fill in the difference between the target ASD stock and the aged stock:


$$\begin{array}{l} N_{0,d,t}=max\left(0,Pop_{t}\times \pi -ASDAged_{t}\right)\times s_{d}, \end{array}$$ (6)

where π = 0.011 in the base case. The term *s*_*d*_ is the ID-status share for group *d*. The aged stock is


$$\begin{array}{l} \text{ASDAge}{\text{d}}_{\text{t}}=\overset{{\text{A}}_{\max}}{\underset{\text{a}=1}{\sum }}\underset{\text{d}\in D}{\sum }{\text{N}}_{\text{a},\text{d},\text{t}}. \end{array}$$

Equation 6 distributes the new entrants by the ID status. Equations 5, 6 keep the aggregate prevalence fixed at the selected value. The age distribution still changes endogenously as cohorts age.

The primary reduced-form stock model does not separately apply age-specific survival probabilities before the age ceiling. Therefore, it represents prevalence-maintaining cohort aging rather than a complete birth-death-migration projection. Individuals exit the primary stock only when they are older than 64. This demographic simplification is treated as a structural limitation.

#### 2.2.2 AI-supported earlier-identification pathway and cost multipliers

The analysis compares BAU with an AI-supported earlier-identification pathway (EIP). EIP is not a specific commercial product. It represents a pathway in which AI-supported screening, triage, referral, or diagnostic support shortens the selected stages of identification and is followed by effective intervention.

The model uses a reduced-form cost-offset structure. Age-specific multipliers represent the net per-person cost effects associated with the EIP. For an individual in age band *k*(*a*), ID-status group *d*, and year *t*, EIP per-person cost is


$$\begin{array}{l} C_{k\left(a\right),d,t}^{EIP}=B_{k\left(a\right),d,t}\times m_{k\left(a\right)}^{eff}, \end{array}$$ (7)

where $m_{k\left(a\right)}^{eff}$ is the effective multiplier for age band *k*(*a*). A value above 1 represents a higher cost than BAU. This reflects an additional intervention investment in early childhood. A value below 1 represents a modeled cost offset in school age or adulthood. Equation 7 defines EIP per-person cost as the BAU cell cost multiplied by the effective age-band multiplier.

Under BAU, the per-person cost remains the age- and ID-specific Buescher cost cell grown forward under Equation 2:


$$\begin{array}{l} C_{k\left(a\right),d,t}^{BAU}=B_{k\left(a\right),d,t}.\; \end{array}$$

The following subsections define the timing and outcome assumptions that produce effective multipliers.

##### 2.2.2.1 Conditional pathway assumptions

This analysis is a calibrated scenario model and not an empirical estimate of the causal effect of current AI deployment. The projected cost pathway comprises three links. AI-supported tools must first shorten screening, triage, referral, or clinician-led assessment. Early identification must lead to timely and effective intervention, and some intervention gains must persist long enough to reduce later support needs.

Prospective studies support the feasibility of selected diagnostic-support and workflow functions (16, 17, 19). They do not estimate the full national pathway through adult costs; therefore, the model treats each downstream link as a scenario assumption. AI-supported tools do not change the underlying ASD prevalence or replace comprehensive clinical assessment. The EIP represents the generalized AI-supported pathways described in the Introduction, rather than any single product. Product-specific costs and effectiveness are not estimated.

##### 2.2.2.2 Identification timing within the 0–5 age band: the ϕ parameter

The model distinguishes identification before age 3 from identification at ages 3–5.^4^

The parameter ϕ∈[0, 1] is the share of children reached through the EIP who are identified before age 3. The remaining share, (1−ϕ), is identified at ages 3–5.

The effective multiplier for age band *k* is


$$\begin{array}{l} m_{k}^{eff}=\phi \cdot m_{k}^{<3}+\left(1-\phi \right)\cdot m_{k}^{3-5}, \end{array}$$ (8)

where superscript < 3 denotes identification before age 3. The superscript ^3 − 5^ denotes identification at ages 3–5.

The base case sets ϕ = 0.60. This is the author-defined timing assumption. It represents a pathway in which the majority of earlier identifications occur through well-child screening or other early referral. The remaining identifications occur later in early childhood. Sensitivity analysis varies ϕ from 0.40 to 0.80.

##### 2.2.2.3 The adult trajectory-shift parameter: ρ

EIBI studies report heterogeneous childhood outcomes. A recent individual-participant-data meta-analysis found clinically significant changes in adaptive behavior, intellectual functioning, and autism severity in some children receiving EIBI. Nevertheless, response varied across children and outcomes (29). Some children show large gains. Others show partial gains or continue to require substantial support.

Historical studies first documented this variation. Lovaas (31) reported that 47% of intensively treated children achieved normal-range intelligence quotient scores and successful first-grade placement. McEachin et al. (32) reported the maintenance of many best-outcome gains into later childhood. Sallows and Graupner (33) reported that 48% of children were rapid learners and were succeeding in regular classrooms at age 7. Howard et al. (41) also reported childhood gains in cognitive, language, and adaptive outcomes.

Adult residential services, direct support, and productivity losses account for a large share of lifetime ASD costs. Therefore, long-run economic results depend on whether some childhood gains persist into lower-support adulthood. Contemporary adult-outcome literature documents substantial variation in independent living, employment, and continuing support needs (34). It does not establish that EIBI by itself directly causes those adult outcomes.

The recent EIBI meta-analysis also does not follow participants into adulthood or estimate adult residential costs. Tsiplova and Ungar (36) identify limited long-term effectiveness data, population heterogeneity, and difficulties measuring costs and outcomes as central challenges in autism economic evaluation. Cost-benefit models provide an additional context, but their long-run savings also depend on assumptions about adult service use (9, 35).

Let ρ∈[0, 1] denote the modeled share of children who receive pre-age-3 EIBI and experience a durable shift toward a lower-support adult cost pathway. It is an author-defined scenario parameter. It is not an observed probability of adult independence.

Neither the historical nor recent literature directly estimates this transition. The intervention studies provide evidence of heterogeneous childhood response. The adult-outcome and economic literatures define the uncertainty surrounding possible long-term pathways. However, they do not determine a single numerical value.

The adult trajectory multiplier for the pre-age-3 subwindow is


$$\begin{array}{l} m_{\text{adult}}^{<3,traj}=\rho \cdot m_{lower}+\left(1-\rho \right)\cdot m_{\text{support}}, \end{array}$$ (9)

where *m*_lower_ = 0.65 represents a lower-support pathway with substantial residual ASD-related costs. The value *m*_support_ = 0.97 represents an adult pathway close to BAU. These are author-defined cost-pathway anchors, not empirically estimated treatment effects.

For identification at ages 3–5, the adult multiplier is specified directly rather than through ρ. The base bundle uses $m_{adult}^{3-5}=0.97$. The Low and High bundles use 0.98 and 0.94, respectively, to represent weaker and stronger residual adult cost offsets for this later-identification sub-window.

The base case sets ρ = 0.35. Equation 10 evaluates Equation 9 at this Base value


$$\begin{array}{l} m_{\text{adult}}^{<3,traj}=0.35\times 0.65+0.65\times 0.97\\ \;=0.2275+0.6305=0.8580. \end{array}$$ (10)

The base value is below the 47%−48% childhood high-response shares reported by Lovaas and Sallows and Graupner. This downward adjustment reflects uncertainty regarding community implementation. It also reflects incomplete intervention dosage and differences between research samples and the national cost population. Recent evidence confirms a heterogeneous childhood response, but it does not provide an observed adult transition probability.

Sensitivity analysis varies ρ from 0.20 to 0.50. A lower value represents a weak conversion of early identification into durable outcomes. The upper value is a favorable scenario near the historical childhood high-response shares. It is not a forecast.

The effective adult multiplier combines the timing and trajectory shift:


$$\begin{array}{l} m_{\text{adult}}^{\text{eff}}=\phi \cdot m_{\text{adult}}^{<3,\text{traj}}+\left(1-\phi \right)\cdot m_{\text{adult}}^{3-5}. \end{array}$$ (11)

##### 2.2.2.4 Evidence for early-childhood and school-age multipliers

Trials and reviews report childhood gains in cognitive, language, adaptive, and social-communication outcomes (23, 24, 28). These outcomes support the direction of the modeled pathway, but they do not provide direct national cost multipliers. Therefore, early-childhood multipliers are treated as scenario assumptions that represent additional intervention spending. The base case sets $m_{0-5}^{<3}=1.20$ for the pre-age-3 subwindow and $m_{0-5}^{3-5}=1.12$ for the ages-3-through-5 sub-window.

The Buescher 0–5 cell already includes services used by young children with ASD in 2011; therefore, it is not a no-intervention counterfactual. Buescher et al. do not identify the share attributable to structured EIBI, ESDM, or other early-intervention services.

Using Buescher's 40/60 ID split, the ID-weighted 0–5 annual cost was approximately $81,120 in 2011 US dollars. Cidav et al. (25) estimated a full clinic-delivered ESDM protocol at approximately $45,580 per child-year in 2015 US dollars. Adding this amount mechanically would imply a multiplier near 1.56. Such an addition would double count services already embedded in the baseline.

The 1.20 base value implies approximately $16,200 in additional annual costs relative to the 2011 Buescher baseline. This is roughly one-third of Cidav's full annual protocol cost. The lower post-age-3 value reflects less time in the 0–5 band for added intervention spending. The Low and High bundles bracket these base assumptions rather than treating them as observed estimates or confidence limits.

The school-age multipliers represent possible later service offsets. Chasson et al. (42) estimated schooling-related savings of up to approximately $208,500 per child over 18 years of schooling. Cooper (43) reported savings of more than $250,000 under some assumptions. Cidav et al. (25) estimated post-intervention ESDM offsets of approximately $19,000 per child-year during school-age follow-up. Piccininni et al. (30) also reported lifetime societal savings from reducing waits for intensive behavioral intervention.

These estimates use different dollar years, time horizons, payer perspectives, and cost categories. However, they cannot be directly inserted into the Buescher all-component cell. Therefore, the model uses attenuated population-average multipliers. The base values are $m_{6-17}^{<3}=0.88$ and $m_{6-17}^{3-5}=\;0.93$.

The Buescher 6–17 ID-weighted annual cost is approximately $65,600 in 2011 US dollars. The base multipliers imply annual reductions of approximately $7,900 and $4,600. These amounts are conservative relative to the published per-child estimates.

Family productivity is another possible factor. Cidav et al. (44) reported substantially lower earnings among mothers of children with ASD. The Buescher school-age cell already includes parental productivity loss. The model does not estimate how much earlier intervention reverses that loss.

##### 2.2.2.5 Construction of the bundled multiplier scenarios

The base component multipliers are literature-informed scenario assumptions calibrated to the published early-intervention costs and later service-offset evidence described above. The Low and High values are author-defined bracketing assumptions around the base specification. They are not estimated treatment effects, confidence limits, or probability bounds. The Low bundle combines lower added spending in the ages-3-through-5 early-childhood component with weaker school-age and adult offsets. The High bundle combines somewhat higher early-childhood spending with stronger later offsets. The pre-age-3 early-childhood component remains 1.20 in all three bundles to preserve the same intervention-cost anchor for children who reach that sub-window. All later multipliers preserve the residual ASD-related costs.

Equation 8 combines the pre-age-3 and ages-3-through-5 components using ϕ = 0.60. For adulthood, Equation 9 first combines *m*_*lower*_ and *m*_*support*_ using the scenario-specific value of ρ; Equation 11 then applies ϕ. Therefore, Table 1 reports the final effective multipliers rather than the raw component assumptions. For example, the base early-childhood value is 0.60(1.20)+0.40(1.12) = 1.168, and the base adult value is 0.60(0.858)+0.40(0.97) = 0.903 after rounding. Supplementary Table S1 provides the complete Low-, Base-, and High-component values and calculation trace.

**Table 1:** Primary bundled earlier-identification pathway scenarios.

Scenario	α_1_ (2025–27)	α_2_ (2028–30)	α_3_ (2031–35)	P^*^(2040+)	ρ	${\text{m}}_{\left(0-5\right)}^{\text{eff}}$	${\text{m}}_{\left(6-17\right)}^{\text{eff}}$	${\text{m}}_{\left(18+\right)}^{\text{eff}}$
Low scenario	1.5%	9.0%	15.0%	35%	0.20	1.160	0.942	0.936
Base scenario	3.0%	18.0%	30.0%	50%	0.35	1.168	0.900	0.903
High scenario	5.0%	25.0%	45.0%	70%	0.50	1.180	0.844	0.862

Low, Base, and High are bundled deployment scenarios rather than one-way sensitivity tests. The three m_eff columns are final scenario-specific effective multipliers used in Equations 13a and 13b; they are derived values, not primitive assumptions. All three incorporate ϕ = 0.60 through Equation 8. The adult value additionally incorporates the scenario-specific ρ through Equations 9 and 11. No further ϕ or ρ adjustment is applied after these values enter the cost calculation. α1, α_2_, and α3 are direct net-reach shares for the stated birth-cohort intervals; P^*^ is the long-run net-reach ceiling from birth year 2040 onward. See Supplementary Table S1 for the underlying component assumptions and the complete Low, Base, and High calculation trace.

#### 2.2.3 Cohort-specific reach of the earlier-identification pathway

Let *p*_*early*_[*b*] denote net EIP reach for birth cohort *b*. It is the share of children with ASD who are correctly identified earlier and gain timely access to effective intervention. It is not a device-use rate, a positive-screen rate, or a pure market-adoption forecast. Instead, net reach reflects losses between technology availability and effective service access. These losses include incomplete deployment, false negatives, indeterminate outputs, referral loss, failure to complete clinical evaluation, reimbursement barriers, and intervention-capacity constraints.

The cohort schedule is


$$p_{\text{early}}\left[b\right]=\{\begin{array}{ll} 0, & b\leq 2024,\\ \alpha_{1}, & 2025\leq b\leq 2027,\\ \alpha_{2}, & 2028\leq b\leq 2030,\\ \alpha_{3}, & 2031\leq b\leq 2035,\\ \alpha_{3}+\left(b-2035\right)\cdot \frac{P*-\alpha_{3}}{5}, & 2036\leq b\leq 2039,\\ P*, & b\geq 2040. \end{array}$$ (12)

The quantities α_1_, α_2_, and α_3_ are net-reach shares for successive birth-cohort intervals. The quantity P^*^ is the long-run plateau. The fifth case linearly interpolates between α_3_ in 2035 and P^*^ in 2040.

The low-reach schedule uses α_1_ = 0.015, α_2_ = 0.09, α_3_ = 0.15, and P^*^ = 0.35. It represents slow deployment and persistent access constraints.

The base schedule uses α_1_ = 0.03, α_2_ = 0.18, α_3_ = 0.30, and P^*^ = 0.50. It represents moderate deployment. The plateau allows for continuing geographic, socioeconomic, reimbursement, and workforce barriers.

The high-reach schedule uses α_1_ = 0.05, α_2_ = 0.25, α_3_ = 0.45, and P^*^ = 0.70. It represents faster deployment and substantial expansion of intervention capacity.

Table 1 combines each reach schedule with the corresponding component-multiplier and trajectory-shift assumptions. Therefore, the Low, Base, and High cases are bundled deployment scenarios rather than reach-only sensitivities. Table 1 reports the final effective age-band multipliers, and Supplementary Table S1 provides their raw component values and arithmetic traces. The early rates represent pilot sites, specialty centers, and initial clinical integration after regulatory clearance. The latter ramp reflects the continuing diffusion and capacity constraints.

Board Certified Behavior Analyst (BCBA) and Board Certified Behavior Analyst-Doctoral job-posting data show a strong unmet demand, whereas geographic studies document county-level gaps in BCBA availability (37, 38). The model does not project workforce supply as a separate stock. Workforce and service capacity enter indirectly through net reach, scenario plateaus, and realized effectiveness, which remains a structural limitation.

The parameters *p*_*early*_[*b*] and ϕ answer different questions. The net reach determines the number of children entering an effective pathway. The timing parameter determines when children are identified within the 0–5 band.

Product performance does not map mechanically into net reach. Jones et al. (10), for example, reported a sensitivity near 81% and specificity between approximately 82 and 90% for eye-tracking case technology. These values provide the performance context, but they are not inserted directly into Equation 12.

False negatives reduce the realized net reach. False positives may result in unnecessary specialist follow-up and additional pressure on scarce services. The model does not estimate these false-positive costs separately, which may understate the total implementation cost. It also holds ϕ constant over time, although clinical protocols and reimbursement could shift identification earlier as deployment matures.

#### 2.2.4 Aggregation of BAU and earlier-identification pathway costs

For projection year *t* and age *a*, birth year is *b* = *t*−*a*. The mean per-person cost under the EIP is a weighted average of the BAU and EIP pathway costs:


$$\begin{array}{l} {\tilde{B}}_{k\left(a\right),d,t}^{EIP}=\left(1-p_{early}\left[b\right]\right)B_{k\left(a\right),d,t}+p_{early}\left[b\right]B_{k\left(a\right),d,t}m_{k\left(a\right)}^{eff}. \end{array}$$ (13a)

Equivalently,


$$\begin{array}{l} {\tilde{B}}_{k\left(a\right),d,t}^{EIP}=B_{k\left(a\right),d,t}\times \left[1+p_{early}\left[b\right]\times \left(m_{k\left(a\right)}^{eff}-1\right)\right].\; \end{array}$$ (13b)

Equation 13b applies the cohort-specific net-reach share to all later ages of that birth cohort. Therefore, children who enter the EIP carry the corresponding multipliers into school age and adulthood.

This is the reduced-form approximation of the 0–5 band. It represents the average cost over the band. It does not assume that identification or intervention begins at birth.

Total national cost under the EIP is


$$\begin{array}{c} \text{TotalCos}{\text{t}}_{\text{t}}^{\text{EIP}}=\overset{{\text{A}}_{\max}}{\underset{\text{a}=0}{\sum }}\underset{\text{d}\in D}{\sum }{\text{N}}_{\text{a},\text{d},\text{t}}\times {\text{B}}_{\text{k}\left(\text{a}\right),\text{d},\text{t}}\times \\ \;\left[1+p_{early}\left[t-a\right]\times \left(m_{k\left(a\right)}^{eff}-1\right)\right]. \end{array}$$ (14)

Equation 14 aggregates EIP costs across all modeled ages and ID-status groups.

The annual difference is


$$\begin{array}{l} {\text{Δ}}_{\text{t}}=\text{TotalCos}{\text{t}}_{\text{t}}^{\text{EIP}}-\text{TotalCos}{\text{t}}_{\text{t}}^{\text{BAU}}. \end{array}$$

A positive Δ_*t*_ indicates higher spending than BAU. It is interpreted as net investment. A negative value indicates annual savings relative to BAU.

The cumulative net present value through year *T* is


$$\begin{array}{c} \text{CumNP}{\text{V}}_{\text{T}}=\overset{\text{T}}{\underset{\text{t}=2025}{\sum }}\frac{\Delta_{\text{t}}}{{\left(1+\text{r}\right)}^{\text{t}-2025}}, \end{array}$$ (15)

where *r* is the real discount rate. The base case uses *r* = 0.03. Sensitivity bounds are 0% and 5%. A negative cumulative value indicates discounted savings relative to BAU.

#### 2.2.5 Illustrative stakeholder-burden allocation

The analysis provides an illustrative distributional allocation across four broad financing and burden groups: Medicaid and other federal programs, state and local education systems, families, and private insurers. These groups are not all payers in the same accounting sense. Families also bear informal caregiving, out-of-pocket expenses, and productivity losses. Buescher et al. report economic costs by category, including medical and non-medical services, special education, residential accommodation, employment support, and productivity losses. Leigh and Du aggregate related service and productivity components for national burden projection (2, 7). Neither study reports national payer-incidence shares. Therefore, the analysis uses an author-defined crosswalk from those economic categories to broad stakeholder groups. The allocation assigns 38% to Medicaid and federal programs, reflecting their role in Medicaid-relevant services, residential care, supportive living, and other public supports; 12% to state and local education systems, reflecting special education costs; 33% to families, reflecting parental productivity loss, caregiving, and out-of-pocket burden; and 17% to private insurers, reflecting privately financed medical, diagnostic, and therapy-related services. The category mapping is literature-informed, but the exact percentages are illustrative assumptions rather than empirically estimated national payer shares.

The allocation is applied only after the model calculates the national BAU and EIP costs. For each year, the same four fixed weights are multiplied by the BAU total, EIP total, and their difference. For example, an incremental national cost or saving of $10 billion would be displayed as $3.8 billion for Medicaid and federal programs, $1.2 billion for education systems, $3.3 billion for families, and $1.7 billion for private insurers. This calculation is a distributional bookkeeping exercise. It does not change the total national costs, scenario rankings, annual crossover dates, or cumulative payback timing.

The fixed-weight allocation does not identify who finances each marginal diagnostic or intervention dollar, who captures each later saving, or how financing responsibility changes across childhood, school age, and adulthood. It does not estimate payer-specific return on investment; transfers among federal, state, local, insurer, and family sectors; or the effects of insurance enrollment changes over time. Near-term diagnostic and intervention costs may be distributed differently from later education, residential, and supportive-care savings. The resulting stakeholder estimates should therefore be interpreted as an illustrative burden distribution, not as a claims-based payer-incidence model. The timing mismatch between early financing and later benefits is interpreted in the Discussion section rather than estimated as a separate market-failure parameter.

Product acquisition, software, maintenance, training, workflow redesign, data infrastructure, and governance costs are not modeled separately. Some may be absorbed within the early-childhood multiplier. Any remaining costs are omitted. This omission may understate the total system cost.

#### 2.2.6 Scenario, sensitivity, and scaling analyses

The primary analysis compares the low, base, and high bundled deployment scenarios, as summarized in Table 1. These scenarios jointly vary the net pathway reach, age-specific cost multipliers, and adult trajectory-shift assumptions. These are not one-way sensitivity tests and do not isolate the marginal effect of any single component.

Two supplementary scaling checks (SC1 and SC2) examine the assumptions that primarily change the magnitude of national dollar totals:

(SC1) ASD prevalence (1.1%, 2.78%, and 3.23%): The base case retains 1.1% for comparability with Leigh and Du. The higher values are the ADDM surveillance estimates for 8-year-old children. They are used as burden-scaling assumptions and not as estimates of direct all-age prevalence.

(SC2) Cost growth rate (3.5%, 4.44%, and 5.5%): This analysis varies the common post-2025 per-person growth rate. The same rate applies to BAU and EIP costs. Therefore, dollar totals are more sensitive than proportional pathway comparisons.

Four deterministic one-way sensitivity analyses examine the assumptions with distinct interpretive roles. In each case, the selected assumption is varied around the base specification, while the remaining assumptions are heldconstant:

- Population growth rate (0.2 and 0.5%): The base case uses 0.2% as a simple approximation of the CBO's long-run outlook. The 0.5% case represents a higher-growth demographic path. Both pathways start from the same 2025 population baseline.
- Discount rate (0%, 3%, and 5%): Early intervention costs occur near the beginning of the horizon. The largest adult cost offsets occur later. Therefore, discounting has a direct effect on cumulative payback.
- Within-band identification timing (ϕ from 0.40 to 0.80): Higher values place more EIP-reached children in the pre-age-3 subwindow. This increases early intervention spending and strengthens the modeled later offsets.
- Adult trajectory shift (ρ from 0.20 to 0.50): This analysis varies the modeled share that moves toward a lower-support adult pathway. It is the most consequential long-run outcome assumption. The upper value is a favorable scenario, not an observed adult effect.

Finally, the unchanged model is extended beyond the primary 2025–2050 horizon through 2060 and 2070 to examine delayed annual crossover and cumulative payback. These are extended-horizon payback checks rather than additional sensitivity analyses.

All scenario, sensitivity, and scaling analyses reported in the main manuscript are deterministic. Supplementary Analysis S4 adds a probabilistic sensitivity analysis (PSA) within the Base deployment scenario. It jointly samples within-band identification timing (ϕ), the adult trajectory-shift parameter (ρ), post-2025 per-person cost growth, and the real discount rate across the ranges used in the deterministic uncertainty analyses. PSA addresses parametric uncertainty under explicit distributional assumptions; it does not resolve the structural or causal uncertainty described below.

## 3 Results

### 3.1 Business-as-usual projection

The business-as-usual (BAU) specification reproduces the Leigh and Du 2025 national cost anchor of $461 billion by construction using Equation 4. Under the maintained assumptions of 1.1% prevalence and 0.2% annual population growth, modeled national ASD-related costs increase to approximately $581.5 billion in 2030, $730.3 billion in 2035, $916.4 billion in 2040, and $1.44 trillion in 2050.

At model initialization, 75.7% of the ASD stock is assigned to the 18+ age band using the Buescher age distribution. The ID-weighted adult cost cell is approximately $65,400 per person in 2011 US dollars. These values describe the concentration of modeled baseline costs in adulthood; they do not establish that earlier identification produces later adult cost reductions.

### 3.2 Primary bundled deployment scenarios

**Figure 1:**
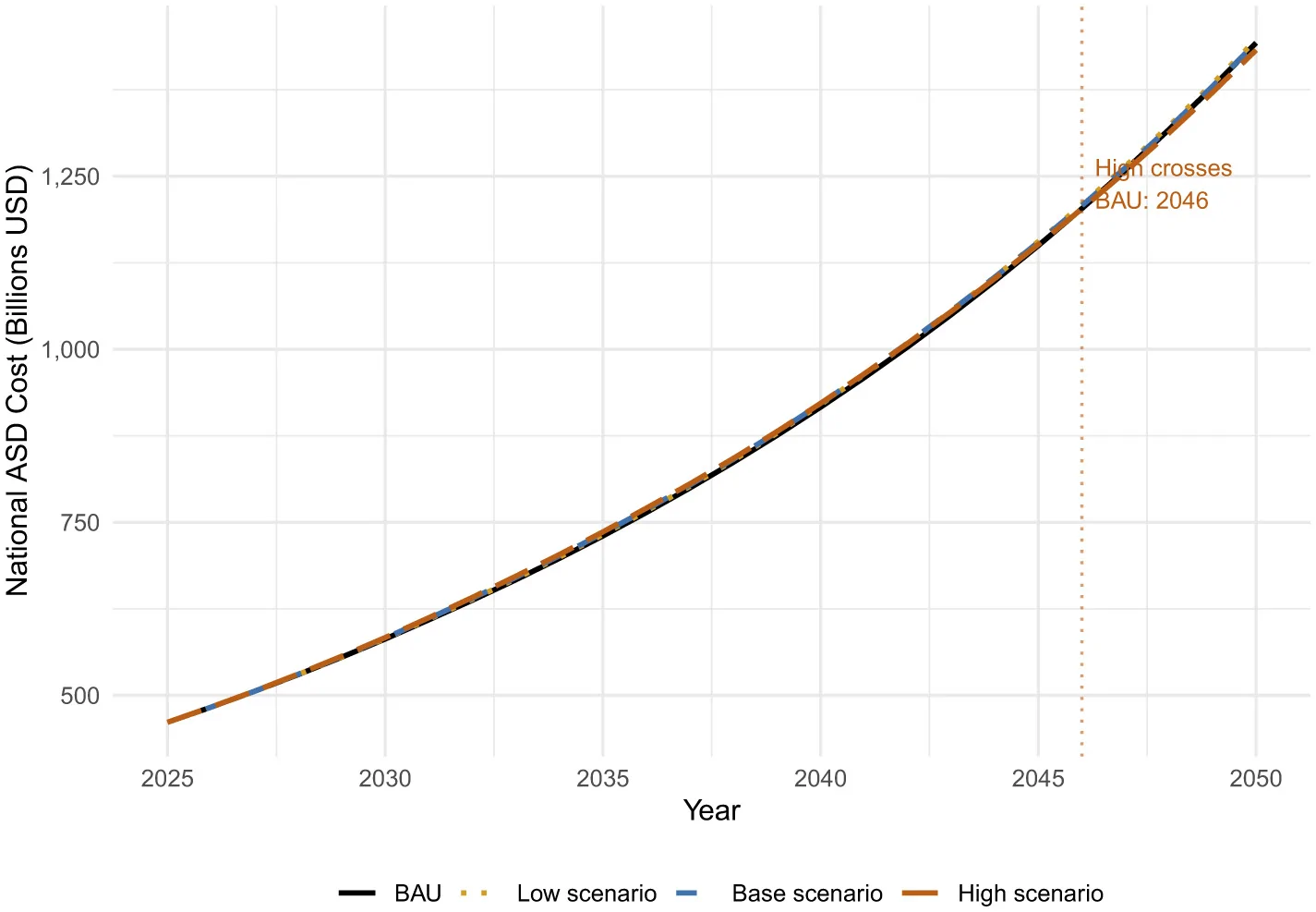
Modeled national ASD cost trajectories, 2025–2050, under BAU and the three bundled earlier-identification pathway scenarios. The high scenario falls below BAU in 2046 under its stated reach, cost-multiplier, and adult trajectory-shift assumptions.

**Figure 2:**
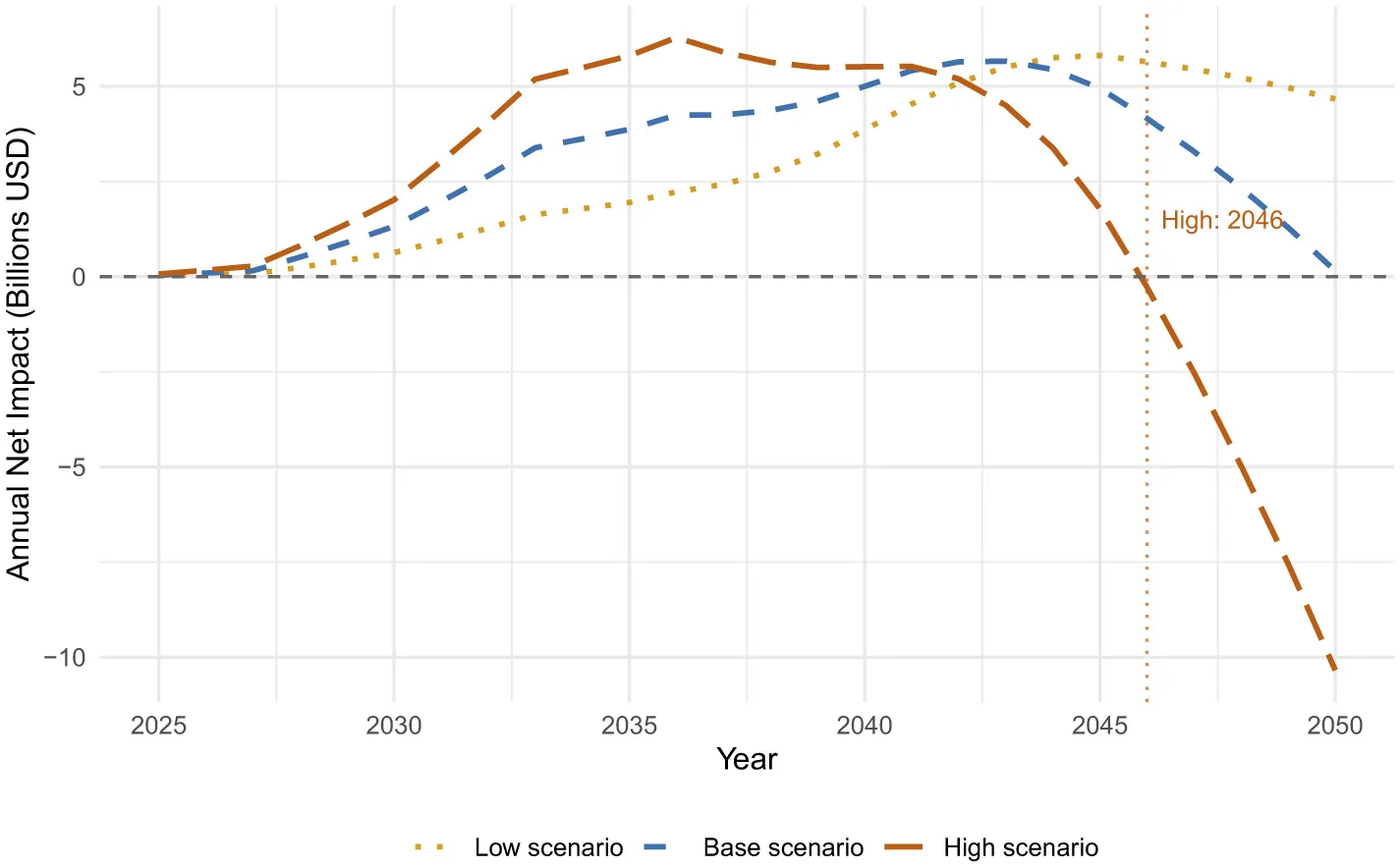
Modeled annual net cost difference (Δ_*t*_ = EIP minus BAU) for 2025–2050. Positive values indicate higher modeled EIP costs, and negative values indicate modeled annual savings relative to BAU.

**Figure 3:**
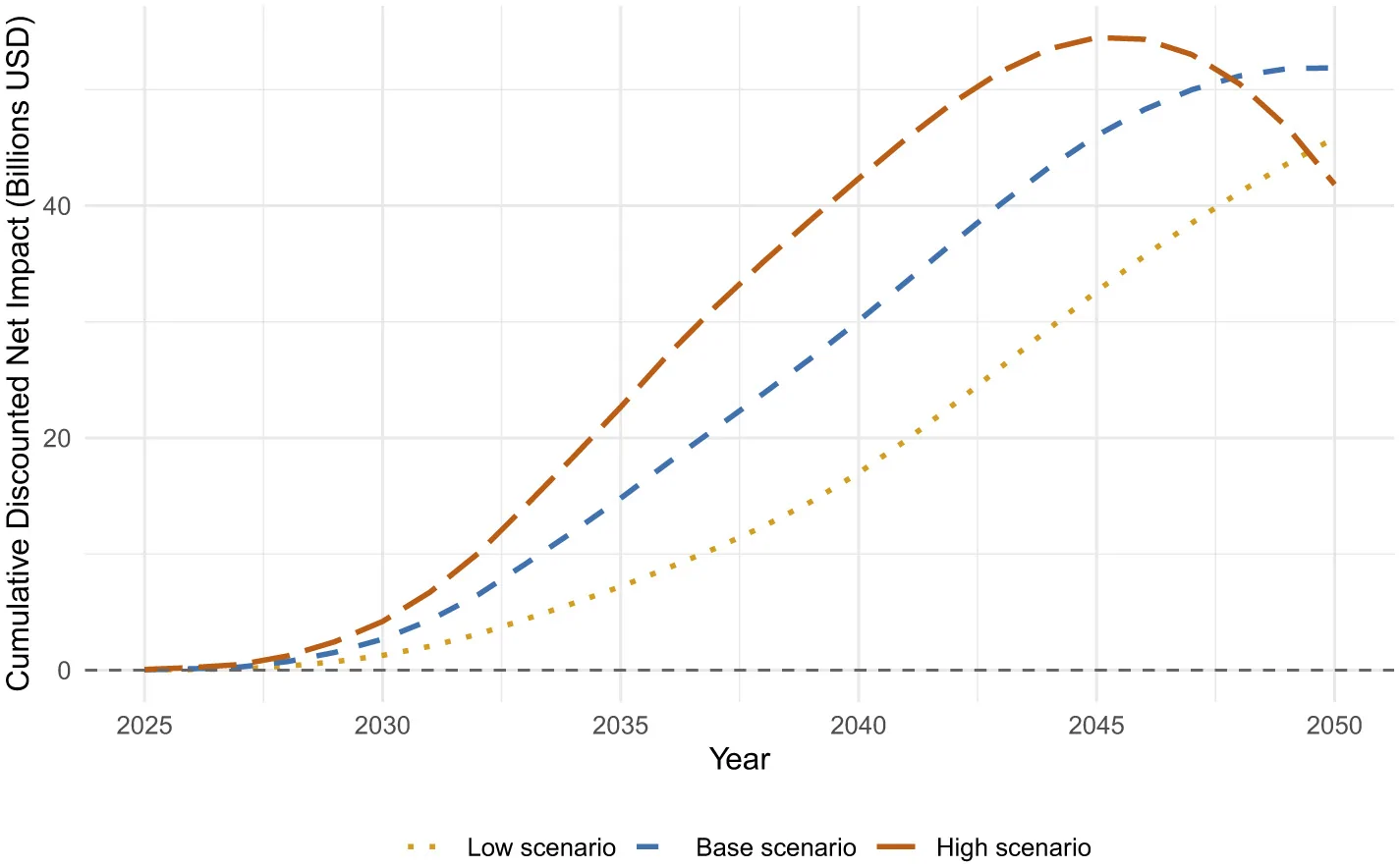
Modeled cumulative discounted net cost difference between EIP and BAU, 2025–2050, using a 3% real discount rate.

Table 1 summarizes the Low, Base, and High deployment scenarios. The three *m*^*eff*^ columns report the final effective age-band multipliers used in the cost calculation, not the underlying pre-age-3 and ages-3-through-5 component assumptions. Each scenario jointly specifies the net pathway reach, these derived effective multipliers, and the adult trajectory-shift parameter. Figure 1 shows the national cost trajectories, Figure 2 shows annual net cost differences, and Figure 3 shows cumulative discounted net cost differences through 2050. Table 2 reports selected-year national costs and net impacts across the three bundled scenarios. The complete component values and arithmetic traces are listed in Supplementary Table S1. Therefore, comparisons across the three scenarios represent differences between complete deployment packages rather than isolated changes in pathway reach or effectiveness.

**Table 2:** Modeled national ASD costs and net impacts by bundled scenario (billions USD).

Year	BAU	EIP Low	EIP Base	EIP High	Net Low	Net Base	Net High	CumNPV Low	CumNPV Base	CumNPV High
2025	461.0	461.0	461.0	461.1	0.0	0.0	0.1	0.0	0.0	0.1
2030	581.5	582.1	582.8	583.5	0.6	1.3	2.0	1.3	2.7	4.2
2035	730.3	732.3	734.2	736.1	1.9	3.9	5.8	7.2	14.8	22.7
2040	916.4	920.3	921.4	921.9	3.9	5.0	5.5	17.0	30.1	42.3
2045	1,149.8	1,155.6	1,154.8	1,151.6	5.8	4.9	1.8	32.6	46.0	54.5
2050	1,442.7	1,447.4	1,442.8	1,432.3	4.7	0.2	-10.3	45.8	51.9	41.8

Net = Δ_*t*_ = EIP minus BAU; positive values indicate higher modeled EIP costs and negative values indicate modeled annual savings. CumNPV is the cumulative discounted net cost difference at a 3 percent real discount rate (Equation 15); negative values indicate modeled cumulative savings.

Because the effective 0–5 multiplier exceeds 1.0 in all three scenarios, the modeled EIP costs initially exceed BAU. Differences between EIP and BAU become more pronounced as the reached cohorts enter the school-age and adult bands, where the scenario multipliers may fall below 1.0.

Under the high deployment scenario, the modeled annual net cost becomes negative in 2046, indicating annual savings relative to BAU under that scenario's assumptions. In 2050, annual net cost is approximately $4.7 billion in the low scenario and $0.2 billion in the base scenario. The high scenario records modeled annual savings of approximately $10.3 billion in 2050.

The cumulative discounted net cost remains positive through 2050 in all three scenarios. The 2050 values are approximately $45.8 billion for the low scenario, $51.9 billion for the base scenario, and $41.8 billion for the high scenario. The high scenario has a lower cumulative net cost than the base scenario by 2050, despite a greater early pathway reach because it also assigns stronger school-age and adult cost offsets. This is a comparison of bundled scenarios and not an estimate of the marginal effect of adoption alone.

### 3.3 Extended-horizon payback checks

**Figure 4:**
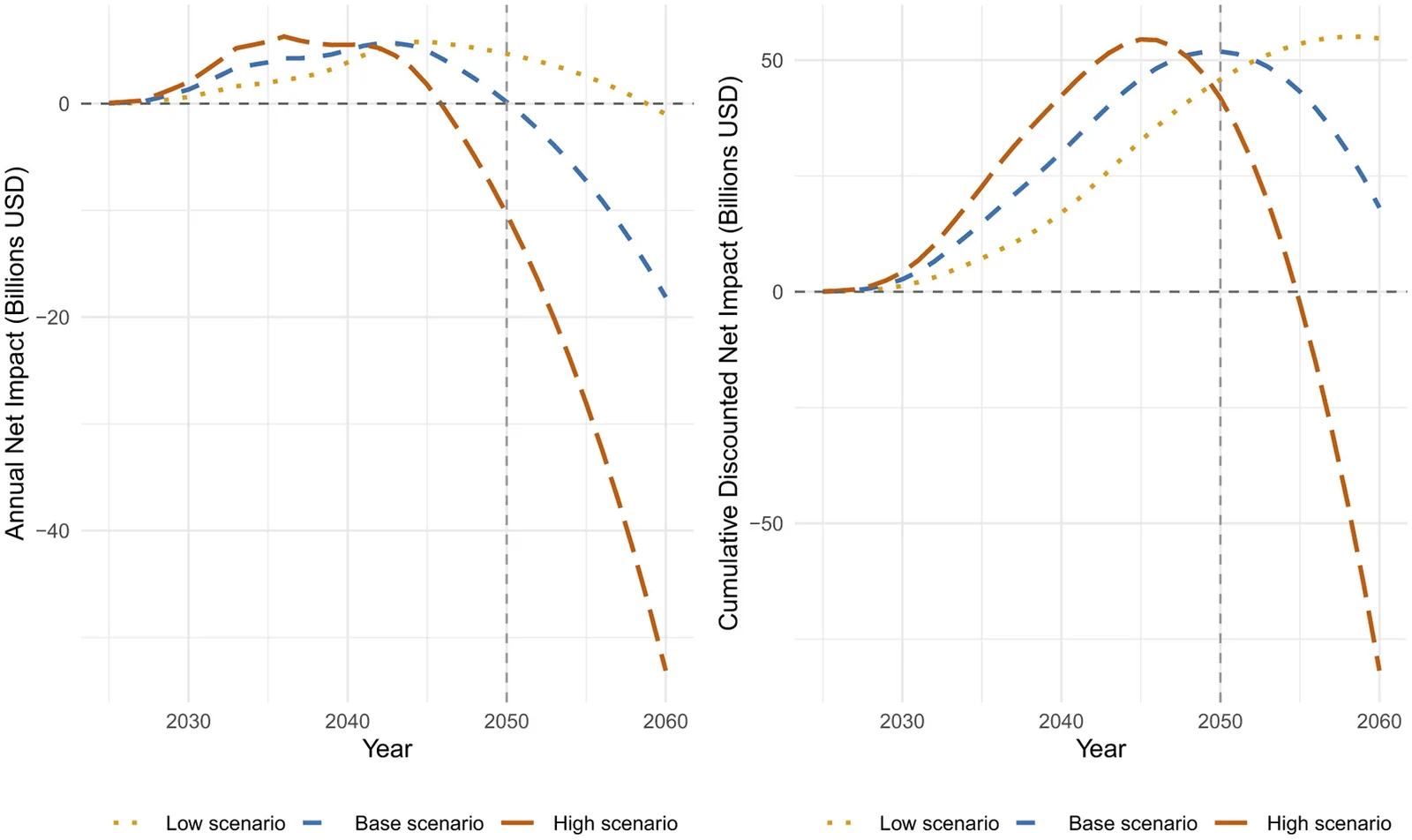
Extended-horizon payback check through 2060 under unchanged scenario assumptions. The **left** panel shows the modeled annual net cost difference, and the **right** panel shows the modeled cumulative discounted net cost difference. The vertical dashed line marks the end of the primary 2025–2050 horizon.

The unchanged model is extended through 2060 to examine payback timing after additional reached cohorts enter adulthood. This extension does not introduce new effectiveness evidence or alter scenario assumptions. Figure 4 presents the annual and cumulative net-cost paths through 2060. Table 3 reports the extended-horizon cost and payback values through 2060.

**Table 3:** Extended-horizon payback check through 2060 under unchanged scenario assumptions (billions USD).

Year	BAU	EIP Low	EIPBase	EIP High	Net Low	Net Base	NetHigh	CumNPV Low	CumNPVBase	CumNPV High
2050	1,442.7	1,447.4	1,442.8	1,432.3	4.7	0.2	−10.3	45.8	51.9	41.8
2055	1,810.1	1,812.7	1,802.9	1,782.1	2.6	−7.2	−28.1	53.5	43.2	−2.4
2060	2,271.2	2,270.2	2,253.0	2,218.1	−1.0	−18.1	−53.1	54.7	18.2	−81.8

This extension uses the same parameters as the primary model and is reported only to assess fiscal payback timing. Net = EIP minus BAU; positive values indicate higher modeled EIP costs and negative values indicate modeled annual savings. CumNPV is the cumulative discounted net cost difference at a 3 percent real discount rate.

Under the high scenario, the modeled cumulative discounted net cost crosses below zero in 2055. The base scenario reaches annual savings in 2051 but remains cumulatively positive through 2060. The low scenario reaches annual savings in 2059 and remains cumulatively positive through 2060.

The supplementary 2070 check shows cumulative payback in 2063 for the base scenario. The low scenario does not achieve cumulative payback by 2070. Supplementary Table S3 reports the 2070 payback check and shows cumulative payback in 2063 for the Base scenario. All crossover and payback dates are conditional on the maintained net-reach, cost-multiplier, and adult trajectory-shift assumptions.

### 3.4 Illustrative stakeholder-burden allocation

Table 4 reports the illustrative stakeholder-burden allocation. Applying fixed illustrative weights to the base scenario's cumulative net cost assigns approximately $19.7 billion to Medicaid and federal programs, $6.2 billion to education systems, $17.1 billion to families, and $8.8 billion to private insurers through 2050. These are arithmetic allocations of the modeled national total. They are not estimates of who finances each marginal intervention dollar, who receives each later saving, or of the payer-specific return on investment.

**Table 4:** Illustrative stakeholder-burden allocation of cumulative net cost, 2025–2050 (billions USD).

Stakeholder group	Illustrative share of BAU costs	Net Low ($B)	Net Base ($B)	Net High ($B)
Medicaid	38%	17.4	19.7	15.9
Education	12%	5.5	6.2	5.0
Families	33%	15.1	17.1	13.8
Private Insurance	17%	7.8	8.8	7.1

Calculated at a 3 percent discount rate. Negative values indicate cumulative savings relative to BAU. The stakeholder shares are author-defined illustrative weights informed by the cost categories in Buescher et al. (2) and Leigh and Du (7). They do not estimate national payer incidence or payer-specific return on investment.

### 3.5 One-way sensitivity analyses

Each one-way analysis changes the stated assumption around the base specification while holding the remaining assumptions constant.

#### 3.5.1 Population growth

Table 5 reports the population-growth sensitivity. Using 0.2% rather than 0.5% annual population growth lowers the modeled 2050 BAU total by approximately 7.4%. The corresponding reductions in the low, base, and high EIP totals are approximately 7.4% to 7.5%. Base-scenario cumulative discounted net cost through 2050 is approximately $51.9 billion under 0.2% growth and $61.7 billion under 0.5% growth. Scenario ordering and crossover years are unchanged.

**Table 5:** One-way sensitivity to population growth (billions USD).

Population growth	BAU 2050 ($B)	EIP Low 2050 ($B)	EIP Base 2050 ($B)	EIP High 2050 ($B)	CumNPV Base ($B)
0.2%/year base	1,442.7	1,447.4	1,442.8	1,432.3	51.9
0.5%/year higher-growth sensitivity	1,558.7	1,564.3	1,559.1	1,546.8	61.7

Both paths begin from the same 2025 population and use the same prevalence, cost growth, pathway reach, and effectiveness assumptions. Relative to the 0.5 percent path, the 0.2 percent base lowers all modeled 2050 pathway totals by approximately 7.4 to 7.5 percent. All non-demographic assumptions are held constant.

#### 3.5.2 Discount rate

Table 6 reports the discount-rate sensitivity. At a 0% discount rate, the modeled cumulative net cost through 2050 is approximately $76.1 billion in the low scenario, $79.3 billion in the base scenario, and $51.8 billion in the high scenario. At 3%, the corresponding values are $45.8 billion, $51.9 billion, and $41.8 billion. At 5%, they are $33.4 billion, $39.9 billion, and $35.7 billion. Because positive annual net costs dominate the primary horizon, applying a higher discount rate reduces the present value of the modeled costs.

**Table 6:** One-way sensitivity to the discount rate (billions USD).

Discount rate	CumNPV Low ($B)	CumNPV Base ($B)	CumNPV High ($B)
0%	76.1	79.3	51.8
3%	45.8	51.9	41.8
5%	33.4	39.9	35.7

Cumulative net cost is calculated over the primary 2025**–**2050 horizon. Negative values indicate modeled cumulative savings relative to BAU. All non-discount assumptions are held at their bundled-scenario values.

#### 3.5.3 Within-band identification timing (**ϕ**)

Table 7 reports the within-band identification-timing sensitivity. Increasing ϕ assigns a larger share of reached children to identification before age 3. Under the maintained base assumptions, this increases the effective 0–5 cost multiplier and lowers the effective school-age and adult multipliers. Modeled cumulative discounted net cost through 2050 rises from approximately $47.7 billion at ϕ = 0.40 to $51.9 billion at ϕ = 0.60 and $56.0 billion at ϕ = 0.80.

**Table 7:** One-way sensitivity to within-band identification timing (ϕ).

Identification-timing case	m___eff (0–5)	m___eff (6–17)	m___eff (18+)	CumNPV base (billions USD)
40% pre-age-3 (referral-triggered)	1.152	0.91	0.925	47.7
60% pre-age-3 (base: 18/24-month visits)	1.168	0.90	0.903	51.9
80% pre-age-3 (systematic 18-month screening)	1.184	0.89	0.880	56.0

Base net-reach values and ρ = 0.35 are held constant. ϕ is the share of EIP-reached children identified before age 3. CumNPV is calculated over the primary 2025**–**2050 horizon at a 3 percent discount rate.

This primary-horizon result reflects the timing of the modeled costs and offsets. More early-intervention spending is included before 2050, while a larger portion of the corresponding adult offsets occurs after the primary horizon. It should not be interpreted as evidence that earlier identification is economically less beneficial.

#### 3.5.4 Adult trajectory-shift parameter (**ρ**)

Table 8 reports the adult trajectory-shift sensitivity. Under the base net-reach and childhood-multiplier assumptions, modeled cumulative discounted net cost through 2050 is approximately $53.1 billion at ρ = 0.20, $51.9 billion at ρ = 0.35, and $50.7 billion at ρ = 0.50. At ρ = 0.50, modeled annual net cost becomes negative in 2050. At the two lower values, the annual net cost remains positive through the primary horizon, and the cumulative net cost remains positive for all three values.

**Table 8:** One-way sensitivity to the adult trajectory-shift parameter ρ.

ρ case	m___eff (18+)	Annual savings begin	Cumulative payback	CumNPV (billions USD)
0.20 (lower trajectory-shift case)	0.932	>2050	>2050	53.1
0.35 (base case)	0.903	>2050	>2050	51.9
0.50 (higher trajectory-shift case)	0.874	2050	>2050	50.7

ρ is the author-defined modeled share of pre-age-3 EIBI-treated children assigned to a durable lower-support adult pathway. Net reach and all other assumptions are held at base values. CumNPV is calculated over the primary 2025**–**2050 horizon at a 3 percent discount rate.

The main effect of ρ appears in the timing of post-2050 payback rather than in large differences in 2050 cumulative totals. These results are conditional on the author-defined adult trajectory-shift mechanism and do not represent the observed adult outcomes following EIBI.

### 3.6 Supplementary scaling checks

Supplementary Table S2 shows the two scaling checks. When prevalence is increased mechanically from 1.1% to the 2022 ADDM value of 3.23%, the modeled BAU cost in 2025 scales from $461 billion to approximately $1.35 trillion, and the base-scenario cumulative net cost through 2050 scales to approximately $152.3 billion. Because the ADDM value is an estimate for 8-year-old children, this calculation is a burden-scaling exercise rather than an all-age prevalence forecast.

Varying the common post-2025 per-person cost-growth rate changes the modeled 2050 BAU total from approximately $1.15 trillion at 3.5% growth to $1.44 trillion at the calibrated rate and $1.86 trillion at 5.5% growth. The base-scenario cumulative net cost through 2050 ranges from approximately $45.8 billion to $59.8 billion. In both scaling checks, the principal effect is on national dollar magnitudes rather than on the structure of the EIP-vs.-BAU comparison.

Supplementary Analysis S4 jointly varies selected continuous parameters within the Base deployment scenario. Across 10,000 draws, the cumulative discounted net cost remains positive through 2050 in every draw. The median annual-savings crossover is 2051, and the median cumulative breakeven year is 2063, matching the deterministic Base timing. These results support the principal Base-scenario pattern under joint parametric uncertainty, while remaining conditional on the fixed Base net-reach schedule and multiplier structure.

### 3.7 Results summary

Across the primary 2025–2050 horizon, the high bundled scenario reaches modeled annual savings in 2046, whereas the base scenario approaches but does not cross the annual breakeven by 2050. The cumulative discounted net cost remains positive through 2050 in all three bundled scenarios. Under the unchanged extended horizon, the high scenario reaches cumulative payback in 2055 and the base scenario in 2063, whereas the low scenario does not reach cumulative payback by 2070. These outcomes remain scenario-dependent and rely on timely pathway access, effective intervention, and the modeled persistence of later cost offsets.

## 4 Discussion

### 4.1 Interpretation of principal findings

This study evaluates AI-supported earlier autism identification through a calibrated, cohort-based scenario model. It does not estimate the observed economic effect of the current national deployment. Across the primary 2025–2050 horizon, all three bundled scenarios remain cumulatively more costly than business as usual (BAU). The High scenario reaches the modeled annual savings in 2046, whereas the Base scenario reaches annual savings in 2051. Under the unchanged extended horizon, the High scenario reaches cumulative payback in 2055 and the Base scenario in 2063. The Low scenario does not reach cumulative payback by 2070. This pattern is best interpreted as early social investment, followed by delayed and assumption-dependent cost offsets.

The economic mechanism does not begin or end with an AI-supported tool. Early identification must lead to completed clinical evaluation, timely intervention access, sufficient intervention intensity, meaningful developmental response, and durable changes in later support needs. A failure at any point weakens the modeled pathway. Limited referral completion reduces the net reach. Inadequate service capacity reduces the realized intervention effects. Weak or non-persistent developmental gains reduce later cost offsets. Therefore, the model evaluates an identification-to-intervention pathway rather than a standalone technology.

Calibration and validation serve different purposes. The BAU model reproduces the 2025 national burden anchor by construction. That calibration provides a transparent baseline for comparing scenarios, but it does not validate the model's future cost trajectories, effective multipliers, or payback dates. Therefore, crossover years should be read as conditional model outputs under the stated assumptions. They do not forecast that a particular technology or health system will achieve those outcomes.

### 4.2 Drivers of scenario results and long-run payback

The Low, Base, and High cases are bundled deployment scenarios. Each combines a net-reach schedule, scenario-specific effective age-band multipliers, and an adult trajectory-shift assumption. The stronger long-run performance of the High scenario cannot be attributed to pathway reach alone. That bundle also assigns larger school-age and adult cost offsets. Conversely, the Low bundle combines a slower reach with weaker downstream offsets. Therefore, comparisons among the bundles show how internally consistent deployment packages perform. They do not identify the marginal effect of one parameter, while all others remain fixed.

The within-band identification parameter, ϕ, changes the timing of modeled spending and offsets. A higher value assigns more children to identification before age 3. This raises early-childhood intervention costs within the primary horizon, while a larger share of the related school-age and adult offsets occurs later. The increase in cumulative net cost through 2050 at higher values of ϕ reflects that timing difference. It should not be interpreted as evidence that identification before age 3 is less valuable. This shows that a horizon ending in 2050 captures early spending more fully than it captures the adult consequences of later birth cohorts.

The adult trajectory-shift parameter, ρ, is the most consequential structural bridge between childhood response and long-run support costs. Historical intervention studies document subsets of children with large childhood gains, while more recent individual-participant evidence confirms that clinically meaningful response is heterogeneous (29, 31, 33). Contemporary adult-outcome evidence also shows wide variation in independent living, employment, and continuing support needs (34). These literatures support examining alternative adult pathways, but they do not estimate the probability needed by this model. They also do not establish that early intensive behavioral intervention (EIBI) by itself causes lower adult residential or supportive-care costs.

For that reason, ρ remains an author-defined scenario parameter rather than an observed transition probability. It represents a possible durable shift toward a lower-support adult cost pathway among a share of children identified before age 3 and receiving effective intervention. A recent methodological study emphasizes the difficulty of translating short-run developmental outcomes into lifetime economic effects when follow-up is limited, and population, interventions, and outcomes are heterogeneous (36). Therefore, the modeled post-2050 payback results are conditional on the persistence and economic relevance of childhood gains.

The remaining uncertainty analyses play different roles. Population growth changes the national scale of projected costs but does not materially alter the pathway mechanism. Discounting changes the present value assigned to early spending and delayed offsets. The prevalence and per-person cost-growth checks primarily scale dollar magnitudes. They do not independently validate the bundled scenario assumptions. This distinction is important because a large national burden can increase both potential costs and offsets without making a particular implementation pathway more effective.

The supplementary PSA complements these deterministic analyses by propagating selected parameter uncertainty jointly within the Base deployment scenario. The results preserve the same broad pattern: positive cumulative net cost through 2050, annual savings around 2051, and cumulative breakeven in the early 2060s. This strengthens confidence that the Base pattern is not an artifact of changing one parameter at a time. It does not validate the assumed identification-to-intervention-to-adult-cost pathway because the net reach and the component multiplier structure remain fixed and the sampling distributions are author-specified rather than empirically estimated.

School-age cost effects may include a family-productivity channel. The Buescher school-age cost cell already incorporates parental productivity losses, and mothers of children with ASD have been shown to experience substantial earnings reductions (44). The effective school-age multipliers may therefore capture some improvement in family economic burden if earlier identification and intervention reduce caregiving demands. However, this model does not estimate that mechanism separately. Consequently, family-productivity benefits could be understated, but adding a separate earnings benefit without suitable data could also double-count costs already contained in the baseline cell.

### 4.3 Implementation capacity, costs, and financing

The principal implementation constraint is service capacity. AI-supported screening, triage, or diagnostic assistance may shorten parts of the identification pathway, but it does not create evaluators, therapists, early-intervention slots, inclusive preschool capacity, or caregiver-support infrastructure. Workforce-demand data, geographic gaps in behavior-analytic services, and reported diagnostic and intervention waitlists indicate that these constraints are substantial (14, 37, 38, 45). The Low scenario is consistent with an environment in which persistent access and capacity barriers limit the completed pathway reach and realized benefits. This should not be interpreted as an empirical estimate of any specific health system.

Implementation also requires resources that are not separately represented in the national cost model. These include device or software acquisition, licensing, training, workflow redesign, interoperability, maintenance, technical support, reimbursement administration, quality assurance, and governance infrastructure. Some costs could be absorbed within existing clinical operations, whereas others would be additional costs. Their size will depend on the technology, setting, scale, and payment arrangement. Future implementation-specific evaluations should measure these costs directly rather than assuming that national service-cost cells fully capture them.

The timing of costs and benefits creates a financing problem. Families, commercial insurers, Medicaid, early-intervention programs, and school systems may bear near-term identification and intervention costs. Some modeled offsets appear much later in education, adult services, residential care, caregiver burden, or public support programs. The organization financing the earlier pathway may therefore capture only part of the eventual benefit. The illustrative stakeholder-burden allocation highlights this potential mismatch, but it is not a claims-based estimate of payer incidence. It also does not identify the marginal payer, beneficiary, or return on investment for any institution.

Several implementation priorities follow in this structure. Reimbursement for validated AI-supported tools should be linked to completed evaluation and timely service access rather than to identification alone. Workforce and service-capacity investments should accompany wider use, particularly in areas with long waits or few specialists. Payment arrangements may also need to recognize that early costs and later benefits occur in different sectors and at different points in the life course. Finally, implementation should include longitudinal measurement of referral completion, intervention uptake, dose, outcomes, and costs. Without those data, neither pathway effectiveness nor payer-specific value can be established.

### 4.4 Clinical role, diagnostic performance, governance, and equity

AI-supported tools should complement, not replace, comprehensive clinician-led assessment. Their appropriate roles may include screening, triage, referral support, structured data collection, and assistance with clinical decision-making. Children with complex presentations, discordant findings, or uncertain results will continue to require professional evaluation. However, this model does not estimate the effectiveness or cost of a specific commercial product. It represents a generalized pathway in which selected stages of identification can be shortened, while clinical responsibility remains with qualified professionals.

Diagnostic performance affects both pathway reach and system burden. False negatives may delay evaluation and reduce the number of children who receive timely intervention. False positives may increase specialist referrals, repeat assessments, family anxiety, and pressure on already constrained services. Indeterminate outputs can create additional testing or follow-up pathways. The model incorporates false negatives and other access losses indirectly through the net pathway reach. It does not separately estimate false-positive costs, unnecessary services, repeat evaluation, referral-queue congestion, or the consequences of indeterminate results (10).

These omissions matter because technical accuracy and operational value are not identical. A tool with favorable average performance may generate limited public health benefits if it is difficult to integrate into clinical workflows, produces burdensome follow-up, or performs unevenly across populations. Implementation studies should therefore report not only sensitivity and specificity but also indeterminate-result rates, referral completion, time to evaluation, time to intervention, clinician workload, and the use of downstream services.

Governance and equity should be treated as core implementation requirements. Existing reviews emphasize external validation, subgroup-performance assessment, bias monitoring, privacy protection, clinician oversight, workflow integration, reimbursement, and accountability (46–48). The original U.S. Food and Drug Administration summary for EarliPoint reported performance differences across participant characteristics, including sex and race or ethnicity, which supports continued subgroup evaluation in broader clinical use (49). Language, culture, insurance coverage, geography, digital access, and local service availability may also affect who completes a pathway.

Equity cannot be assessed from identification performance alone. An AI-supported tool may increase or accelerate identification while widening disparities if underserved families cannot obtain confirmatory evaluation or intervention. Conversely, tools embedded in primary care or community settings could improve access if they are externally validated, culturally and linguistically appropriate, supported by clinician oversight, and linked to available services. Public health value therefore depends on both fair technical performance and equitable downstream capacity.

### 4.5 Limitations and research priorities

This study has several structural limitations. It is a scenario model rather than a causal evaluation of the observed deployment. The effective age-band multipliers are reduced-form assumptions that summarize several possible changes in service use, education, caregiver burden, and adult support costs. The bundled scenarios vary several components together and therefore do not identify the independent effect of pathway reach, intervention intensity, or adult trajectory change. The model also assigns the full adult trajectory-shift mechanism only to the pre-age-3 subwindow. Children identified at ages 3–5 years may experience meaningful and durable benefits that are only partially represented.

The adult pathway is the most important and unresolved source of uncertainty. The value of ρ is not estimated from long-term follow-up of children entering AI-supported pathways. The lower-support adult multiplier is also an author-defined cost-pathway anchor rather than an empirically observed national average. The model preserves substantial residual costs and does not assume the elimination of support needs, but exact adult offsets remain uncertain. Consequently, the 2060 and 2070 analyses should be interpreted as payback checks under unchanged assumptions and not as precise long-run forecasts.

This model omits several implementation processes. It does not separately track true positives, false positives, false negatives, indeterminate outputs, referral queues, intervention-slot constraints, treatment adherence, intervention dose, or variations in implementation fidelity. Product acquisition, software, training, maintenance, workflow, interoperability, reimbursement, and governance costs are not independently estimated. Workforce expansion is represented indirectly through net reach and realized effectiveness rather than through a separate supply model. These omissions may either increase costs or reduce realized offsets relative to the modeled scenarios.

Demographic and costing assumptions are also simplified. The primary cohort stock does not separately parameterize mortality before the age of 65, and the maximum modeled age is 64. Population growth is represented through constant-rate paths rather than a complete birth-death-migration projection. Prevalence is held constant within each scenario, although the measured prevalence and identification practices may change. A common cost-growth rate is applied across healthcare, education, residential care, wages, and productivity, even though these categories may follow different paths. The illustrative stakeholder allocation is a fixed bookkeeping crosswalk rather than a payer-incidence model.

The analysis focuses on monetary burden and does not value many outcomes that matter to people with autism and their families. It omits quality of life, communication, adaptive functioning, educational participation, caregiver wellbeing, family mental health, and the intrinsic value of receiving an accurate and timely explanation of developmental needs. It also does not separately estimate changes in caregiver employment or earnings. These omissions mean that societal value may be understated. At the same time, monetizing them without appropriate data could introduce double counting or speculative benefits.

The next empirical step is not simply to estimate another national cost total. Prospective studies should follow the full pathway from AI-supported identification through referral completion, clinician-led evaluation, intervention access, treatment dose, and developmental response. They should report subgroup performance and equity for both identification and service access. A longer follow-up period should measure school service use, family outcomes, adult support needs, employment, residential services, and payer-specific costs. Linking these data would allow future models to replace author-defined pathway parameters with observed transition rates and test whether short-run developmental changes translate into durable economic effects.

### 4.6 Interpretation and qualifications

This study's contribution is a transparent national framework for examining how alternative identification-to-intervention pathways could affect future societal costs. It is not an evaluation of a specific commercial technology, an estimate of realized national savings, or evidence that early identification alone changes adult outcomes. The model identifies the assumptions and system conditions under which earlier pathway access can produce delayed cost offsets. Those conditions include reliable clinical integration, timely and effective intervention, sufficient workforce capacity, equitable access, appropriate financing, and durable changes in later support needs. Therefore, the findings should inform planning and empirical study design rather than be treated as a forecast of exact savings or payback dates.

## 5 Conclusion

This study provides a calibrated cohort-based framework for examining how AI-supported earlier autism identification could affect future US societal costs under alternative identification-to-intervention pathways. The estimates are conditional scenario projections rather than the observed effects of the current national deployment. All three bundled scenarios remain cumulatively more costly than business-as-usual by 2050. In the Base scenario, the cumulative discounted net cost is approximately $51.9 billion over 2025–2050. The High scenario reaches the modeled annual savings in 2046 and cumulative payback in 2055. The Base scenario reaches annual savings in 2051 and cumulative payback in 2063. The Low scenario does not reach cumulative payback by 2070.

These outcomes depend on more than the technical performance of an AI-supported tool. Earlier identification must lead to completed clinical evaluation, timely and effective intervention, adequate workforce and service capacity, and durable changes in later support needs. Implementation must also preserve clinician responsibility, address diagnostic error and workflow burden, protect privacy, monitor subgroup performance, and ensure that families can obtain downstream services on which the modeled benefits depend. Therefore, the findings do not support the general claim that AI-supported identification will save money. They identify the conditions under which pathway acceleration could produce delayed economic offsets and broader social value.

AI-supported earlier identification can function as public health infrastructure only when it is embedded in a capable and equitable identification-to-intervention system. The model offers a transparent basis for planning, comparing implementation assumptions, and identifying the evidence required for stronger economic evaluation. Prospective studies should follow children from AI-supported identification through referral completion, intervention access and dose, developmental and family outcomes, use of adult support, and lifetime costs. Such evidence is necessary before the exact national savings, payback timing, or payer-specific returns can be established.

## Data Availability

The original contributions presented in the study are included in the article/supplementary material, further inquiries can be directed to the corresponding author.

## Footnotes

1 Sohl et al. (19) was a prospective observational study without a concurrent control group.

2 The available evidence remains heterogeneous, and the cited studies do not establish population-wide causal effects or support replacing comprehensive clinician-led evaluation. Detailed implementation, validation, equity, and safety considerations are addressed in the Discussion.

3 An annual rate of 0.5% was used as the baseline demographic assumption in the originally submitted manuscript. In the revised analysis, it is retained as a sensitivity case rather than the base case.

4 EarliPoint provides one practical example of a technology that spans both sub-windows. Its initial FDA clearances covered children aged 16–30 months. Its current indication extends from 16–95 months, thereby including children in both modeled sub-windows (18, 50, 51).
