# Supplementary material for: AI-supported early autism identification as public health infrastructure: economic implications for the United States using a scenario-based economic model
Source: Front Public Health. 2026 Aug 17;14:1886106. doi: 10.3389/fpubh.2026.1886106 (PMC13526990; doi:10.3389/fpubh.2026.1886106)
Supplement: Supplementary file 1 [file Supplementary_file_1.docx]

Supplementary Material

Supplementary Tables S1-S3 and Supplementary Analysis S4

# Supplementary Table S1: Core Model Parameters and Bundled Multiplier Calculation Trace

## S1A. Core parameter grid

***Supplementary Table S1A. Core Model Parameters and Their Roles in Bundled Scenarios, One-Way Sensitivities, and Scaling Checks***

| **Parameter** | **Base Value** | **Source or Derivation** | **Scenario, Sensitivity, or Scaling Values** |
| --- | --- | --- | --- |
| ASD prevalence (BAU main model) | 1.1% | Leigh-Du/Buescher comparability assumption | Scaling check (SC1): 2.78% and 3.23% (8-year-old ADDM estimates) |
| ID share | 40% ID, 60% non-ID | Buescher/Leigh-Du assumption | Fixed in reported analyses |
| Age distribution of ASD cases | Buescher 2011 age shares | Calculated from Buescher et al. (2014) U.S. age-band counts | Fixed in reported analyses |
| Per-person annual costs (2011 USD) | Buescher et al. (2014): 6 cells | Buescher et al. (2014) annual U.S. cost cells by age band and ID status | Fixed published cells |
| ID-weighted 0–5 annual cost | ~$81,120 | 0.4 x $107,863 + 0.6 x $63,291 | Derived; not varied separately |
| ID-weighted 6–17 annual cost | ~$65,599 | 0.4 x $85,690 + 0.6 x $52,205 | Derived; not varied separately |
| ID-weighted 18+ annual cost | ~$65,402 | 0.4 x $88,026 + 0.6 x $50,320 | Derived; not varied separately |
| 2025 calibration aggregate K | ~$251.0B | 2025 ASD stock at 1.1% prevalence and a rounded 342M population (U.S. Census Bureau 2026), evaluated at 2011 Buescher costs | Derived calibration aggregate |
| Per-person cost growth g | 4.44%/yr | Analytic calibration to the rounded Leigh-Du 2025 target of $461B | Scaling check (SC2): 3.5% and 5.5% after 2025 |
| Calibration method | g = (461e9/K)^(1/14) - 1 | Closed-form calibration equation (4) | Fixed calibration method |
| Maximum modeled age | 64 years | Model implementation; pre-65 mortality is not separately parameterized | Fixed at 64 in reported analyses |
| Time horizon | Primary: 2025–2050; payback checks: 2060 and 2070 | Primary policy horizon plus unchanged-model payback extensions | 2060 and 2070 extended-horizon checks |
| Discount rate for cumulative net present value | 3% real | Model assumption for social present-value calculations | One-way sensitivity: 0% and 5% |
| Population growth rate (base) | 0.2%/yr | Constant-growth approximation to the CBO long-run demographic outlook | One-way sensitivity: 0.5%/yr |
| Net-reach share alpha1 (2025–2027) [Base] | 3.0% | Base bundled deployment scenario | Bundled scenarios: 1.5% (Low), 3.0% (Base), 5.0% (High) |
| Net-reach share alpha2 (2028–2030) [Base] | 18.0% | Base bundled deployment scenario | Bundled scenarios: 9.0% (Low), 18.0% (Base), 25.0% (High) |
| Net-reach share alpha3 (2031–2035) [Base] | 30.0% | Base bundled deployment scenario | Bundled scenarios: 15.0% (Low), 30.0% (Base), 45.0% (High) |
| Net-reach plateau P* (2040+) [Base] | 50% | Base bundled deployment scenario | Bundled scenarios: 35% (Low), 50% (Base), 70% (High) |
| Early-intervention multiplier, 0–5, pre-age-3 | 1.20 | Scenario assumption informed by early-intervention cost evidence | Fixed at 1.20 across bundled scenarios |
| Early-intervention multiplier, 0–5, ages 3–5 | 1.12 | Scenario assumption for identification at ages 3–5 | Bundled scenarios: 1.10 (Low), 1.12 (Base), 1.15 (High) |
| School-age multiplier, 6–17, pre-age-3 | 0.88 | Scenario assumption informed by school-age cost-offset evidence | Bundled scenarios: 0.93 (Low), 0.88 (Base), 0.82 (High) |
| School-age multiplier, 6–17, ages 3–5 | 0.93 | Scenario assumption for identification at ages 3–5 | Bundled scenarios: 0.96 (Low), 0.93 (Base), 0.88 (High) |
| Lower-support adult multiplier (m_lower) | 0.65 | Author-defined lower-support adult pathway | Fixed at 0.65 |
| Adult support multiplier within the pre-age-3 trajectory equation (m_support) | 0.97 | Author-defined near-BAU adult support pathway | Fixed at 0.97 within the pre-age-3 trajectory equation |
| rho: modeled durable adult trajectory-shift share [Base] | 0.35 | Author-defined scenario parameter informed by heterogeneous childhood response and adult-outcome uncertainty | One-way sensitivity and bundles: 0.20–0.50 |
| Adult multiplier, pre-age-3 trajectory at rho=0.35 | 0.858 | rho*m_lower + (1-rho)*m_support | 0.906 at rho=0.20; 0.858 at 0.35; 0.810 at 0.50 |
| Adult multiplier, ages 3–5 [Base] | 0.97 | No full adult trajectory shift assigned to the ages-3-through-5 sub-window | Bundled scenarios: 0.98 (Low), 0.97 (Base), 0.94 (High) |
| phi: share of EIP-reached children identified before age 3 | 0.60 | Author-defined identification-timing assumption | One-way sensitivity: 0.40–0.80 |
| Effective m(0–5) at phi=0.60 [Base] | 1.168 | phi*m(<3) + (1-phi)*m(3-5) | Bundles: 1.160–1.180; phi sensitivity: 1.152–1.184 |
| Effective m(6–17) at phi=0.60 [Base] | 0.900 | phi*m(<3) + (1-phi)*m(3-5) | Bundles: 0.844–0.942; phi sensitivity: 0.890–0.910 |
| Overall m(18+) at phi=0.60 and rho=0.35 [Base] | 0.903 | phi*m(<3) + (1-phi)*m(3-5) | Bundles: 0.862–0.936; rho sensitivity: 0.874–0.932; phi sensitivity: 0.880–0.925 |
| *Note: Source-derived values are taken from or calculated from published cost cells. Author-defined values are scenario assumptions. Bundled scenario ranges should not be interpreted as one-way sensitivity ranges.* | | | |

## S1B. Calculation trace for the effective multipliers reported in main-text Table 1

***Supplementary Table S1B. Calculation Trace for the Effective Multipliers Reported in Main-Text Table 1***

| **Scenario** | **Age Band** | **Component Calculation** | **m_eff** |
| --- | --- | --- | --- |
| Low | 0–5 | 0.60(1.20) + 0.40(1.10) | 1.160 |
| Low | 6–17 | 0.60(0.93) + 0.40(0.96) | 0.942 |
| Low | 18+ | 0.60[0.20(0.65)+0.80(0.97)] + 0.40(0.98) | 0.936 |
| Base | 0–5 | 0.60(1.20) + 0.40(1.12) | 1.168 |
| Base | 6–17 | 0.60(0.88) + 0.40(0.93) | 0.900 |
| Base | 18+ | 0.60[0.35(0.65)+0.65(0.97)] + 0.40(0.97) | 0.903 |
| High | 0–5 | 0.60(1.20) + 0.40(1.15) | 1.180 |
| High | 6–17 | 0.60(0.82) + 0.40(0.88) | 0.844 |
| High | 18+ | 0.60[0.50(0.65)+0.50(0.97)] + 0.40(0.94) | 0.862 |

# Supplementary Table S2: Scaling Checks

## S2A. SC1: ASD prevalence

***Supplementary Table S2A. Supplementary Scaling Check (SC1): ASD Prevalence***

| **Prevalence** | **BAU 2025 ($B)** | **BAU 2050 ($B)** | **CumNPV Base ($B)** |
| --- | --- | --- | --- |
| 1.1% (Leigh-Du baseline) | 461 | 1,443 | 51.9 |
| 2.78% (ADDM 2020, 1-in-36 among 8-year-olds) | 1,165 | 3,646 | 131.1 |
| 3.23% (ADDM 2022, 1-in-31 among 8-year-olds) | 1,354 | 4,236 | 152.3 |
| *Note: All values scale linearly with prevalence while age distribution, ID composition, net reach, and cost multipliers are held constant. The ADDM estimates apply to 8-year-old children and are used only as burden-scaling values, not as direct all-age prevalence estimates.* | | | |

## S2B. SC2: Post-2025 per-person cost growth

***Supplementary Table S2B. Supplementary Scaling Check (SC2): Post-2025 Per-Person Cost Growth***

| **Cost Growth Rate** | **BAU 2025 ($B)** | **BAU 2050 ($B)** | **CumNPV Base ($B)** |
| --- | --- | --- | --- |
| 3.50%/yr post-2025 | 461 | 1,151 | 45.8 |
| 4.44%/yr base | 461 | 1,443 | 51.9 |
| 5.50%/yr post-2025 | 461 | 1,858 | 59.8 |
| *Note: All rows preserve the calibrated Leigh-Du 2025 BAU anchor of $461 billion. Alternative rates are applied only after 2025 and affect BAU and EIP costs through the same common growth factor.* | | | |

# Supplementary Table S3: Extended-Horizon 2070 Payback Check

***Supplementary Table S3. Extended-Horizon Payback Check Through 2070 (Billions USD)***

| **Year** | **BAU** | **EIP Low** | **EIP Base** | **EIP High** | **Net Low** | **Net Base** | **Net High** | **CumNPV Low** | **CumNPV Base** | **CumNPV High** |
| --- | --- | --- | --- | --- | --- | --- | --- | --- | --- | --- |
| 2,050 | 1,442.7 | 1,447.4 | 1,442.8 | 1,432.3 | 4.7 | 0.2 | -10.3 | 45.8 | 51.9 | 41.8 |
| 2,060 | 2,271.2 | 2,270.2 | 2,253.0 | 2,218.1 | -1.0 | -18.1 | -53.1 | 54.7 | 18.2 | -81.8 |
| 2,070 | 3,575.4 | 3,560.8 | 3,519.3 | 3,437.3 | -14.6 | -56.2 | -138.1 | 32.6 | -90.7 | -364.0 |
| *Note: This appendix case extends the same model assumptions to 2070. Net = EIP minus BAU; positive values indicate higher modeled EIP costs and negative values indicate modeled annual savings. CumNPV is the cumulative discounted net cost difference at a 3 percent real discount rate.* | | | | | | | | | | |

# Supplementary Analysis S4: Probabilistic Sensitivity Analysis

## S4.1 Rationale and scope

The main analysis uses deterministic bundled scenarios, one-way sensitivities, scaling checks, and extended-horizon payback checks. This supplementary probabilistic sensitivity analysis (PSA) evaluates whether the principal Base-scenario pattern is preserved when selected continuous parameters are varied jointly rather than one at a time. It complements the deterministic analysis and does not replace the Low, Base, and High scenario comparisons.

The PSA addresses parametric uncertainty within the Base deployment scenario. It does not test the causal validity of the identification-to-intervention-to-adult-cost pathway, assign probabilities to the Low, Base, or High deployment scenarios, or resolve uncertainty about future technologies, service-delivery systems, reimbursement, or policy. Those structural and causal limitations remain as discussed in the main text.

## S4.2 Methods

Four parameters were assigned triangular distributions. The minimum and maximum match the bounds already used in the manuscript's deterministic uncertainty analyses, and the mode equals the Base value. Triangular distributions were chosen because they transparently preserve the stated bounds while placing the greatest density near the Base specification.

***Supplementary Table S4.1. Parameters Jointly Sampled in the Base-Scenario PSA***

| **Parameter** | **Symbol** | **Distribution** | **Deterministic Source** |
| --- | --- | --- | --- |
| Within-band identification timing | phi | Triangular (0.40, 0.60, 0.80) | phi one-way sensitivity |
| Adult trajectory shift | rho | Triangular (0.20, 0.35, 0.50) | rho one-way sensitivity |
| Post-2025 per-person cost growth | g | Triangular (3.50%, 4.438%, 5.50%) | SC2 cost-growth scaling check |
| Real discount rate | r | Triangular (0%, 3%, 5%) | discount-rate one-way sensitivity |
| *Note: Values are listed as minimum, mode, and maximum. The calibrated cost-growth mode is approximately 4.438 percent. Parameters are sampled independently because empirical correlation estimates are unavailable.* | | | |

Net pathway reach, ASD prevalence, population growth, component cost multipliers, the adult cost-pathway anchors, the ID split, and the age-64 boundary remain at their Base values. Net reach and component multipliers define the deterministic deployment bundles rather than empirically estimated probability distributions. The higher-prevalence cases are burden-scaling checks based on child surveillance values rather than an all-age uncertainty distribution. Population growth has a one-sided higher-growth check rather than a three-point probability specification. Holding these elements fixed avoids assigning unsupported probabilities to structural scenario choices.

The four sampled parameters are treated as independent because no empirical joint distribution is available. This independence assumption and the triangular distributional forms are additional probabilistic-analysis assumptions, but no new model value or causal pathway is introduced. Each draw uses the same cohort cost engine, Base net-reach schedule, and component multipliers as the deterministic model.

Ten thousand Monte Carlo draws were evaluated through 2070 using a fixed seed and a cross-platform Park-Miller pseudorandom stream. For each draw, the model recorded cumulative discounted net cost in 2050, 2060, and 2070, the first year of annual net savings, and the first year of cumulative breakeven. Discounting was recalculated using each draw's sampled real discount rate. If a draw did not reach a milestone by 2070, its year was coded as 2071 for the rank-based analysis; no draw required that code in the completed simulation. Parameter influence was summarized with partial rank correlation coefficients (PRCCs).

At the Base point (phi = 0.60, rho = 0.35, post-2025 cost growth of approximately 4.438 percent, and a 3 percent real discount rate), the PSA wrapper reproduces the deterministic Base results exactly. This validates the probabilistic wrapper against the manuscript cost engine; it does not constitute external validation of the underlying causal assumptions.

## S4.3 Results

***Supplementary Table S4.2. Base-Scenario PSA Results Across 10,000 Draws***

| **Outcome** | **Deterministic Base** | **PSA Median** | **Central 95% Simulation Interval** |
| --- | --- | --- | --- |
| CumNPV 2050 ($B) | 51.9 | 54.3 | 41.5 to 74.9 |
| CumNPV 2060 ($B) | 18.2 | 16.9 | -9.9 to 27.6 |
| CumNPV 2070 ($B) | -90.7 | -106.1 | -327.4 to -29.5 |
| Annual-savings crossover year | 2051 | 2051 | 2050 to 2051 |
| Cumulative breakeven year | 2063 | 2063 | 2060 to 2066 |
| *Note: Positive CumNPV denotes cumulative net investment relative to BAU; negative CumNPV denotes cumulative savings. The deterministic Base uses a 3 percent real discount rate. Each PSA draw uses its sampled discount rate. Intervals are central simulation intervals under the stated triangular distributions and are not empirical confidence intervals.* | | | |

Cumulative breakeven is not reached by 2050 in any draw. The proportion of draws reaching cumulative breakeven is 7.9 percent by 2060 and 100.0 percent by 2070. Annual net savings are reached by 34.4 percent of draws by 2050 and by 100.0 percent by 2060. The latest cumulative breakeven year among the completed draws is 2069.

**Supplementary Figure S4.** (A) Distribution of cumulative discounted net cost in 2050, with the deterministic Base result marked. (B) Cumulative proportion of PSA draws reaching breakeven by year. (C) Partial rank correlation coefficients for cumulative breakeven year.

***Supplementary Table S4.3. Partial Rank Correlations for Selected PSA Outcomes***

| **Parameter** | **CumNPV 2050** | **Annual Crossover Year** | **Cumulative Breakeven Year** |
| --- | --- | --- | --- |
| Identification timing (phi) | 0.82 | -0.53 | -0.69 |
| Adult trajectory shift (rho) | -0.38 | -0.77 | -0.89 |
| Post-2025 cost growth (g) | 0.92 | 0.00 | -0.71 |
| Real discount rate (r) | -0.99 | -0.01 | 0.93 |
| *Note: For year outcomes, a negative coefficient means that a higher parameter value moves the milestone earlier. PRCCs describe monotonic association within the specified PSA and should not be interpreted causally.* | | | |

## S4.4 Interpretation

The PSA supports the principal deterministic Base-scenario pattern under the selected joint parameter ranges. Cumulative net cost remains positive through 2050 in every draw, with a median of 54.3 billion dollars and a central 95 percent simulation interval of 41.5 to 74.9 billion dollars. By 2070, cumulative net cost is negative in every draw. The median annual-savings crossover remains 2051, and the median cumulative breakeven year remains 2063, matching the deterministic Base timing.

The influence pattern also agrees with the deterministic interpretation, with an important qualification. The real discount rate and post-2025 cost growth have the largest absolute associations with cumulative net cost in 2050, while the adult trajectory parameter has a smaller influence on that within-window total. The adult trajectory shift is the strongest driver of the annual-savings crossover and the second strongest driver of cumulative breakeven timing after the discount rate. Identification timing and cost growth also materially affect cumulative payback timing.

These findings strengthen the conclusion that the Base scenario represents near-term net investment followed by delayed and assumption-dependent offsets. They do not demonstrate that the assumed pathway will occur in practice. The PSA conditions on the Base net-reach schedule and multiplier structure, and its simulation intervals reflect the chosen distributions rather than empirical sampling uncertainty. Structural and causal uncertainty therefore remains the principal limitation.
